# Concurrent inhibition of pBADS99 synergistically improves MEK inhibitor efficacy in KRAS^G12D^-mutant pancreatic ductal adenocarcinoma

**DOI:** 10.1038/s41419-024-06551-7

**Published:** 2024-02-26

**Authors:** Yan Qin Tan, Bowen Sun, Xi Zhang, Shuwei Zhang, Hui Guo, Basappa Basappa, Tao Zhu, Gautam Sethi, Peter E. Lobie, Vijay Pandey

**Affiliations:** 1 Institute of Biopharmaceutical and Health Engineering and Tsinghua Berkeley Shenzhen Institute, Tsinghua Shenzhen International Graduate School, Tsinghua University, Shenzhen, 518055 People’s Republic of China; 2 Food Science and Technology Program, Department of Life Sciences, BNU-HKBU United International College, Zhuhai, 519087 Guangdong People’s Republic of China; 3 Shenzhen Bay Laboratory, Shenzhen, 518055 Guangdong People’s Republic of China; 4 Laboratory of Chemical Biology, Department of Studies in Organic Chemistry, University of Mysore, Manasagangotri, 570006 Mysore India; 5 Department of Oncology, The First Affiliated Hospital of USTC, Center for Advanced Interdisciplinary Science and Biomedicine of IHM, Division of Life Sciences and Medicine, University of Science and Technology of China, Hefei, Anhui 230027 People’s Republic of China; 6 Hefei National Laboratory for Physical Sciences, University of Science and Technology of China, Hefei, Anhui 230027 People’s Republic of China; 7 Department of Pharmacology, Yong Loo Lin School of Medicine, National University of Singapore, Singapore, 117600 Singapore; 8 NUS Centre for Cancer Research, Yong Loo Lin School of Medicine, National University of Singapore, Singapore, 117599 Singapore

**Keywords:** Drug development, Targeted therapies, Pancreatic cancer, Drug screening

## Abstract

Therapeutic targeting of KRAS-mutant pancreatic ductal adenocarcinoma (PDAC) has remained a significant challenge in clinical oncology. Direct targeting of KRAS has proven difficult, and inhibition of the KRAS effectors have shown limited success due to compensatory activation of survival pathways. Being a core downstream effector of the KRAS-driven p44/42 MAPK and PI3K/AKT pathways governing intrinsic apoptosis, BAD phosphorylation emerges as a promising therapeutic target. Herein, a positive association of the pBADS99/BAD ratio with higher disease stage and worse overall survival of PDAC was observed. Homology-directed repair of BAD to BADS99A or small molecule inhibition of BADS99 phosphorylation by NCK significantly reduced PDAC cell viability by promoting cell cycle arrest and apoptosis. NCK also abrogated the growth of preformed colonies of PDAC cells in 3D culture. Furthermore, high-throughput screening with an oncology drug library to identify potential combinations revealed a strong synergistic effect between NCK and MEK inhibitors in PDAC cells harboring either wild-type or mutant-KRAS. Mechanistically, both mutant-KRAS and MEK inhibition increased the phosphorylation of BADS99 in PDAC cells, an effect abrogated by NCK. Combined pBADS99-MEK inhibition demonstrated strong synergy in reducing cell viability, enhancing apoptosis, and achieving xenograft stasis in KRAS-mutant PDAC. In conclusion, the inhibition of BADS99 phosphorylation enhances the efficacy of MEK inhibition, and their combined inhibition represents a mechanistically based and potentially effective therapeutic strategy for the treatment of KRAS-mutant PDAC.

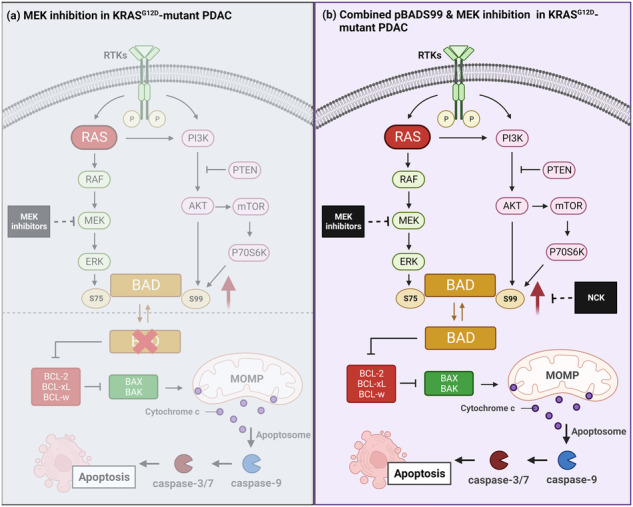

## Introduction

Pancreatic ductal adenocarcinoma (PDAC) is characterized by a meagre 5-year survival rate of approximately 10% [1]. Dismally, late-stage diagnosis excludes the majority of PDAC patients (>80%) from the option of surgery, thereby facilitating disease metastasis, recurrence, and mortality [2, 3]. In accordance with NCCN guidelines, FOLFIRINOX stands as the primary systemic therapy for PDAC, offering improved survival outcomes yet accompanied by a higher rate of adverse events when compared to Gemcitabine [4, 5]. Associated with over 90% of PDAC cases, v‐Ki‐ras2 Kirsten rat sarcoma viral oncogene homolog (KRAS) mutation (~50% KRAS^G12D^ subtype) constitutes an initiating event that impels the transitions from pancreatic intraepithelial neoplasia (PanIN) to pancreatic oncogenesis, followed by subsequent mutations of TP53, CDKN2A, and SMAD4 [6]. Despite a well-delineated genetic and functional profile, KRAS has long been deemed a challenging “undruggable target,” a notion that persisted until recent FDA approvals of drugs Sotorasib [7] and Adagrasib [8], which target the KRAS^G12C^ mutation in treating non-small cell lung cancer (NSCLC). Nevertheless, the KRAS^G12C^ mutation only accounts for 1% of all KRAS mutations within PDAC [9], confining the benefits of selective KRAS^G12C^-targeted therapy to a very limited portion of PDAC patients [10]. Furthermore, KRAS inhibition has been associated with drug resistance due to the activation of rescue mechanisms promoting PDAC cell survival, rendering PDAC patients with few viable therapeutic options. The pursuit of effective strategies centered on targeting novel oncogenic drivers has emerged as a critical necessity to improve the prognosis of PDAC patients.

The aberrant activation of the p44/42 MAPK pathway, also known as RAS-RAF-MEK-ERK signaling, in KRAS-driven cancers, has generated significant interest in targeting the downstream effectors of this cascade as a therapeutic approach [11–13]. In this context, the inhibition of p44/42 MAPK activity achievable through MEK inhibition was explored as a potential strategy for cancers carrying KRAS mutations, drawing from their efficacy in treating BRAF^V600^-mutant melanoma [14, 15]. However, various clinical trials have yielded disappointing results, failing to demonstrate significant survival benefits of MEK inhibition, either as a single-agent treatment or combined with chemotherapy for KRAS-mutant cancers [16–18]. The efficacy of MEK inhibition encounters substantial challenges, including rapid drug resistance stemming from p44/42 MAPK reactivation [19] or the simultaneous activation of parallel pathways such as PI3K/AKT [20, 21], STAT3 [22] and Hippo [23] signaling. Central to the intrinsic apoptosis process, BAD emerges as a pivotal downstream effector protein commonly regulated by both the p44/42 MAPK and PI3K/AKT pathways [24, 25]. Phosphorylation of human BAD at Serine (S) 75 is predominantly dependent on the p44/42 MAPK pathway, whereas phosphorylation at S99 and S118 is primarily mediated by the PI3K/AKT pathway [24]. Notably, BAD may also undergo phosphorylation at S75, S99, and S118 by mitochondrial PKA [26–28] and PKC-iota [29]. Consequently, aberrant activation of p44/42 MAPK and PI3K/AKT pathways drives site-specific phosphorylation of BAD, promoting the survival of cancer cells [24, 25]. Indeed, abundant research underscores the substantial impact of the PI3K/AKT pathway on the response of KRAS-mutant cancer to MEK inhibition [30, 31] and the efficacy of concurrently targeting both the MAPK and PI3K/AKT signaling pathways has been demonstrated [32, 33].

This study investigated the concurrent inhibition of two parallel pathways within KRAS-mutant PDAC, specifically co-targeting MEK and phosphorylation of S99 in BAD. The investigation provides mechanistic and preclinical evidence that substantiates the potential therapeutic effects of this approach for treating KRAS-mutant cancers.

## Results

### A higher pBADS99/BAD ratio in PDAC is positively correlated with more advanced disease stage and worse overall survival in patients

**Fig. 1:**
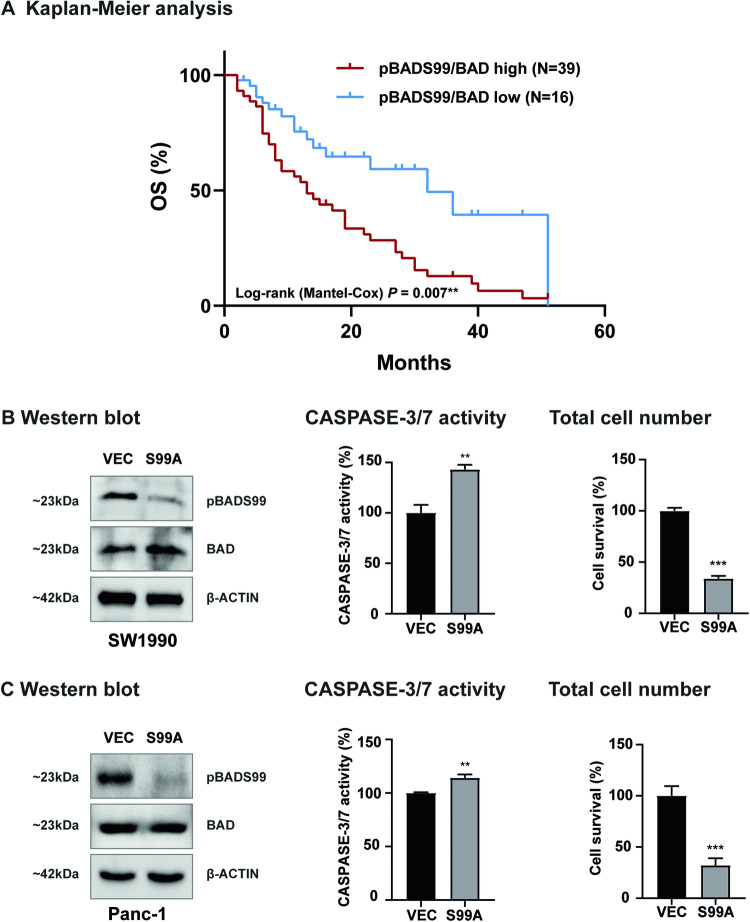
A higher pBADS99/BAD ratio in PDAC is positively correlated with higher disease stage and worse overall survival in patients with PDAC. **A** Kaplan–Meier analysis of overall survival (OS) in PDAC patients stratified according to pBADSer99/BAD low or high expression in PDAC tissues. **B** Western blot analysis of pBADS99 and BAD protein levels in SW1990 cells after transfection with a pBADS99A knock-in plasmid (S99A) or vector control (VEC) for 8 hours (h). The sizes of detected protein bands in kDa are shown on the left. Corresponding CASPASE-3/7 activity and cell survival of SW1990 cells after transfection are shown on the right. Data represent means ± SD (*n* = 3). *P < 0.05, **P < 0.01, and ***P < 0.001. **C** Western blot analysis of pBADS99 and BAD protein levels in Panc-1 cells after transfection with a pBADS99A knock-in plasmid (S99A) or vector control (VEC) for 8 h. The sizes of detected protein bands in kDa are shown on the left. Corresponding CASPASE-3/7 activity and cell survival of Panc-1 cells after transfection are shown on the right. Data represent means ± SD (*n* = 3). *P < 0.05, **P < 0.01, and ***P < 0.001.

Increased phosphorylation of human BAD at S75 (murine S112) and S99 (murine S136) residue has been reported to be associated with worse survival outcomes in patients with TNBC and OC [34, 35]. Therefore, IHC analysis was utilized to determine the phosphorylation of BAD at either the S75 or S99 residues in specimens of PDAC, as described in the methodology section. As observed in Table 1, higher levels of pBADS99/BAD were positively associated with the diameter of the tumor and higher TNM disease stage. Higher levels of pBADS75/BAD were positively correlated with age but negatively correlated with grade, TNM, and distant metastasis (SI 1). Next, a potential association between the pBADS99/BAD ratio and the overall survival of PDAC patients was determined. Using a *log-rank test* analysis, a significant negative correlation of a higher ratio of pBADS99/BAD in PDAC specimens with decreased overall survival (OS) (*P* < 0.01) was observed (Fig. 1A). No significant correlation (*P* = 0.891) was observed between the pBADS75/BAD ratio and OS in PDAC specimens (SI 2). Thus, the pBADS99/BAD ratio predicts poor survival outcomes in PDAC patients.

**Table 1:** Correlation analysis between pBADS99/BAD levels and clinicopathological features of PDAC patients.

Cohort	Total (N)	High (%)	Low (%)	*P-value*
Gender				>0.999
Male	24	71	29	
Female	31	71	29	
Age				>0.999
<60	21	71	29	
>=60	34	71	29	
Diameter				0.006**
<=4	33	64	36	
>4	22	82	18	
Grade				0.455
1	6	67	33	
2	16	75	25	
3	33	70	30	
TNM				0.004**
I	24	62.5	37.5	
II	15	80	20	
III	6	83	17	
IV	10	70	30	
Lymph node metastasis				0.876
Yes	18	72	28	
No	37	70	30	
Distant metastasis				0.539
Yes	9	67	33	
No	46	72	28	

*P < 0.05, **P < 0.01, and ***P < 0.001. An immunoreactive score (IRS) ≥2 was categorized as high pBADS99/BAD and an IRS <2 was categorized as low pBADS99/BAD in the PDAC patient cohort.

Subsequently, by using a *CRISPR-Cas9* approach to perform homology-directed repair (HDR) of BAD to BADS99A, it was demonstrated that the reduction in pBADS99/BAD in SW1990 or Panc-1 cells resulted in increased CASPASE 3/7 activity and decreased cell survival (Fig. 1B, C, SI 3A-B). Hence, pBADS99 is a potential therapeutic target for PDAC.

### Pharmacological inhibition of pBADS99 in PDAC cells induces G0/G1 cell cycle arrest and promotes apoptotic cell death

**Fig. 2:**
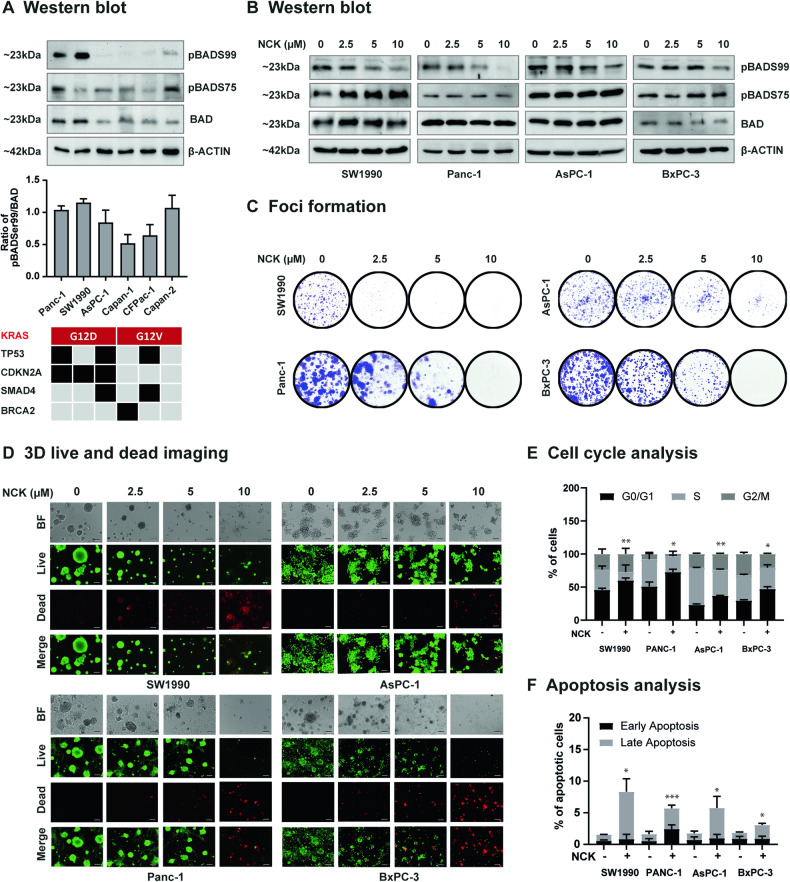
Pharmacological inhibition of pBADS99 in PDAC cells induces G0/G1 cell cycle arrest and promotes apoptotic cell death. **A** Western blot analysis was used to assess the level of BAD proteins in KRAS-mutant PDAC cells (Panc-1, SW1990, AsPC-1, Capan-1, CFPac-1 and Capan-2). β-ACTIN was used as input control. The sizes of detected protein bands in kDa are shown on the left. The densitometric analysis of protein blots is shown below along with the mutational status of the PDAC cell lines. Oncogene (red): KRAS; Tumor suppressor genes (black): TP53, CDKN2A, SMAD4 and BRCA2. **B** Western blot analysis was used to assess the level of BAD protein in PDAC cells (SW1990, Panc-1, AsPC-1, and BxPC-3) after treatment with 0-10 μM of NCK for 72 h. β-ACTIN was used as input control. The sizes of detected protein bands in kDa are shown on the left. **C** Crystal violet staining of foci in colonies of PDAC cells after exposure to 0-10 μM of NCK for 9 days. **D** Microscopic visualization of Calcein-AM (green) stained colonies (live) and BOBO-3 Iodide (red) stained cell debris (dead) generated by PDAC cells cultured in 3D Matrigel after exposure to 0-10 μM of NCK for 12 days. Scale bars, 100 μm. BF: Bright-field image; Merge: Merged image of Live and Dead. **E**. Flow cytometry analysis of PI staining for cell cycle state of PDAC cells measured after treatment with 0-10 μM of NCK for 48 h using flow cytometry analysis as described in materials and methods. Data represent means ± SD (*n* = 3). *P < 0.05, **P < 0.01, and ***P < 0.001. **F**. Flow cytometry analysis of Annexin-V and propidium iodide (PI) staining of apoptotic cell death of PDAC cells measured after treatment with 0-10 μM of NCK for 72 h using flow cytometry analysis as described in materials and methods. The upper left quadrant (Annexin V−, PI + ) represents cell debris, the upper right quadrant (Annexin V + , PI + ) represents late apoptosis, the lower right quadrant (Annexin V + , PI-) represents the early apoptosis and the lower left quadrant (Annexin V-, PI-) represented live cells. Data represent means ± SD (*n* = 3). *P < 0.05, **P < 0.01, and ***P < 0.001.

NCK, a derivative of NPB (an inhibitor of pBADS99 [36]) identified to possess enhanced efficacy in inhibiting pBADS99 [37] was used herein to assess the impact of pharmacological inhibition of BADS99 on PDAC cells (both KRAS-mutant and wild-type). The inhibitory concentration 50% (IC_50_) values of NCK for PDAC cells in total cell number assays are summarized in Table 2 and SI 4. Specifically, KRAS^G12D^-mutant PDAC cells demonstrated an IC_50_ of less than ~2 μM NCK, whereas KRAS^G12V^-mutant PDAC cells exhibited an IC_50_ of less than ~4 μM NCK. KRAS wild-type BxPC-3 cells displayed an IC_50_ of ~1.74 μM NCK. In contrast, immortalized human pancreatic duct epithelial cells (HPDE) (Table 2), exhibited an IC_50_ for NCK of 64.4 μM. A positive correlation between a higher endogenous ratio of pBADS99/BAD and lower IC_50_ values of NCK in PDAC cells was observed (Table 2, Fig. 2A). Notably, the most prevalent KRAS mutation, KRAS^G12D^ was associated with the lowest IC_50_ values for NCK among PDAC cell lines, specifically SW1990, Panc-1, and AsPC-1, which exhibited a higher pBADS99/BAD ratio (Table 2, Fig. 2A).

**Table 2:** IC_50_ values of NCK in *immortalized* pancreatic duct epithelial and PDAC cell lines.

	Cell line	Oncogene		Tumor suppressor gene				NCK IC_50_ ± SD (μM)
		KRAS	BRAF	TP53	BRCA2	CDKN2A	SMAD4	
PDAC	SW1990	**↑ G12D**				**↓**		0.91 ± 0.23
	Panc-1	**↑ G12D**		**↓**		**↓**		1.96 ± 0.47
	AsPC-1	**↑ G12D**		**↓**		**↓**	**↓**	2.20 ± 0.27
	CFPac-1	**↑ G12V**		**↓**			**↓**	2.17 ± 0.10
	Capan-1	**↑ G12V**			**↓**			1.43 ± 0.17
	Capan-2	**↑ G12V**						4.03 ± 0.61
	BxPC-3		**↑**	**↓**		**↓**	**↓**	1.74 ± 0.18
Immortalized Normal	HPDE6-C7							64.36 ± 25.48

**↑** represents gain-of-function mutation, while **↓** represents loss-of-function mutation.

Subsequently, NCK treatment of KRAS^G12D^-mutant PDAC cells SW1990, Panc-1, AsPC-1, and KRAS wild-type BxPC-3 cells exhibited decreased ratios of pBADS99/BAD as demonstrated using western blot analysis (Fig. 2B, SI 5A). NCK treatment produced a significant dose-dependent decrease in the capacity of PDAC cell lines to form foci in monolayer culture (Fig. 2C, SI 5B). Notably, amongst all cell lines, SW1990 cells displayed the greatest response to NCK treatment in terms of attenuation of foci-forming capacity (SI 5B). Also, NCK treatment resulted in a dose-dependent suppression of 3D growth of preformed PDAC cell colonies in Matrigel (Fig. 2D, SI 5C), as demonstrated by increased red fluorescence in the Live-Dead assay, indicative of apoptotic cells stained by BOBO-3, and decreased green fluorescence, representing live cells stained by Calcein-AM.

Furthermore, the effect of NCK on the cell cycle and apoptosis of PDAC cells was examined. NCK treatment induced G0/G1 growth arrest (Fig. 2E, SI 5D) and triggered apoptotic cell death (Fig. 2F, SI 5E) across all four PDAC cell lines (SW1990, Panc-1, AsPC-1 and BxPC-3). Notably, SW1990, characterized by the highest pBADS99/BAD ratio (as per Fig. 2A) among PDAC cell lines, exhibited the highest sensitivity among these cell lines to the pro-apoptotic effects of NCK treatment (Fig. 2F, SI 5E). These findings indicated that pharmacological inhibition of pBADS99 by NCK reduces cell viability by inducing G0/G1 cell cycle arrest and promoting apoptotic cell death in PDAC cells, with the most pronounced effect observed in cells expressing a high ratio of pBADS99/BAD.

### High-throughput drug screening demonstrated that Trametinib synergizes with NCK in AsPC-1 and BxPC-3 cell line

**Fig. 3:**
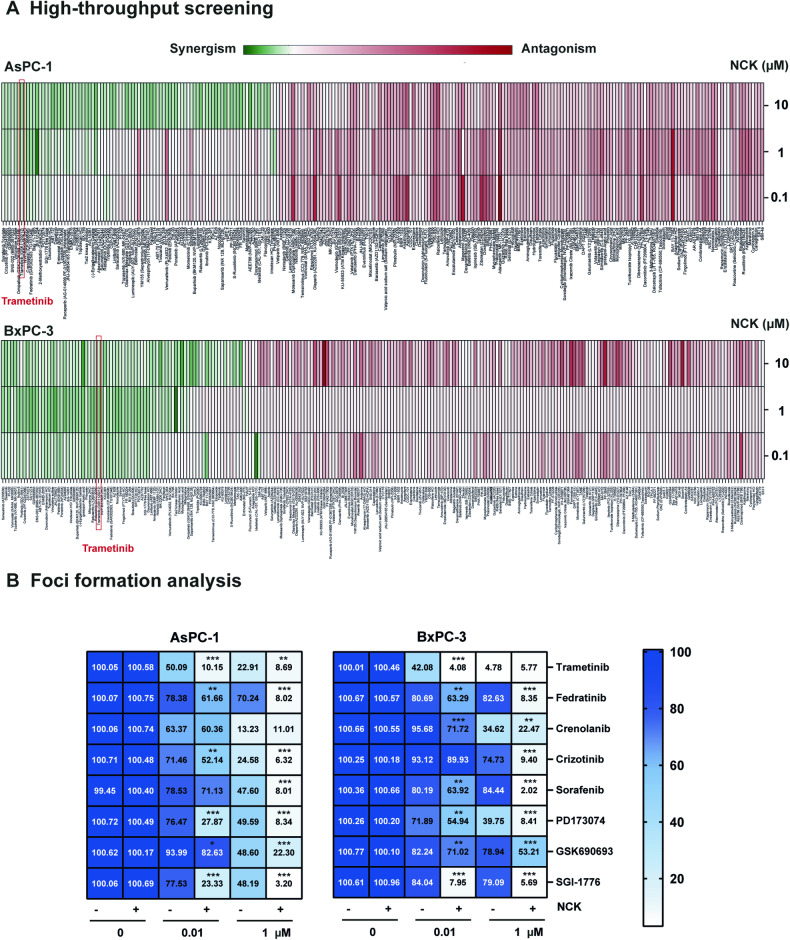
High-throughput drug screening demonstrates that Trametinib synergizes with NCK in AsPC-1 and BxPC-3 cell lines. **A**. Heatmap plot depicts combination index (CI) of all 247 compounds in combination with NCK in AsPC-1 and BxPC-3 cells obtained by high-throughput screening. CI was calculated using bliss independence method (CI= (E_A_ + E_B_-E_A_E_B_)/E_AB_), where CI < 1 denotes synergistic interaction and CI > 1 denotes antagonistic interaction. **B** Heatmap plot depicts quantification of crystal violet staining of foci in colonies of AsPC-1 and BxPC-3 cells after exposure to 8 compounds (at 0, 0.01 and 1 μM) synergistic with NCK in the high-throughput drug screening of AsPC-1 and BxPC-3 cells. Data represent the ratio of cell viability relative to the vehicle (mean, *n* = 3). *P < 0.05, **P < 0.01, and ***P < 0.001.

To explore the therapeutic potential of NCK-based synergistic combinations for the treatment of PDAC, high-throughput drug screening with the Cambridge Cancer Compound Library was utilized [38]. High-throughput drug screening was performed in combination with 247 anticancer agents with increasing doses of NCK for the treatment of AsPC-1 and BxPC-3 cells as described in the methodology section (SI 6-7). AsPC-1 was selected because in addition to its KRAS^G12D^ mutation, it also harbors mutation of TP53, CDKN2A and SMAD4 [39], four main genetic alterations that drive PDAC; [40] whereas BxPC-3 was selected as a KRAS wild-type, but TP53, CDKN2A and SMAD4 mutant PDAC cells [39] to ensure that the combinations obtained exhibits synergistic effect in PDAC regardless of KRAS-mutational status (Table 2). Among the 247 compounds, compounds targeting protein tyrosine kinase, JAK/STAT, PI3K/AKT, MAPK, cell cycle, cytoskeletal signaling, epigenetics or DNA damage were observed to synergistically decrease cell viability of AsPC-1 and BxPC-3 cells at all three log doses (0.1,1, and 10 μM) of NCK (Fig. 3A, SI 8). Upon further analysis using the Combination Index (CI), Trametinib emerged as the compound with the most pronounced synergistic effect when combined with NCK in both PDAC cell lines (Table 3, SI 9). These findings were corroborated by the results of the foci formation assay, further supporting the CI analysis (Fig. 3B, SI 10-13). Consequently, the data suggested that the MEK inhibitor, Trametinib, synergizes effectively with NCK and decreases cell viability of AsPC-1 or BxPC-3 cells.

**Table 3:** CI_0.5-0.8_ of the combined NCK-compound treatment in PDAC cells.

	CI_0.5-0.8_	AsPC-1	BxPC-3
**NCK** +	**Trametinib**	0.344 ± 0.022	0.150 ± 0.030
	**Fedratinib**	0.420 ± 0.017	0.168 ± 0.004
	**Crenolanib**	0.420 ± 0.051	0.240 ± 0.011
	**Crizotinib**	0.449 ± 0.074	0.288 ± 0.026
	**Sorafenib**	0.529 ± 0.004	0.532 ± 0.015
	**PD173074**	0.547 ± 0.032	0.572 ± 0.071
	**GSK690693**	0.590 ± 0.130	1.008 ± 0.024
	**SGI-1776**	0.953 ± 0.110	1.123 ± 0.048

### NCK synergizes with MEK inhibitors to decrease KRAS-mutant PDAC cell survival

**Fig. 4:**
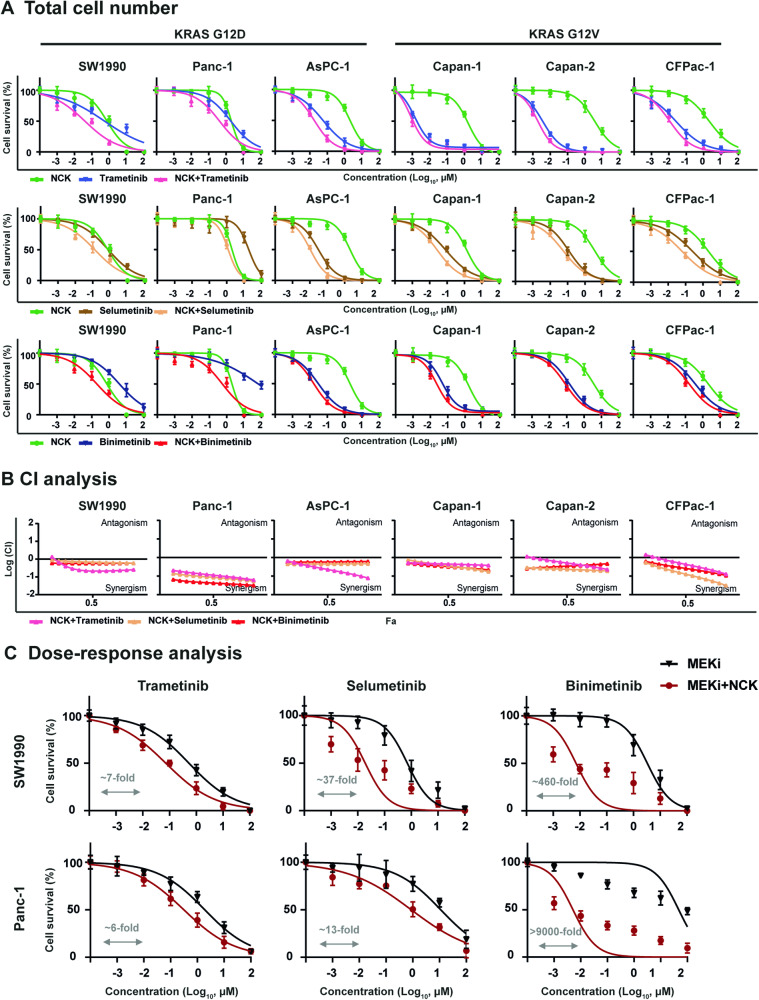
NCK synergizes with MEK inhibitors to decrease KRAS-mutant PDAC cell survival. **A** Total cell number assay was performed to measure the survival fraction of PDAC cells after treatment with indicated concentration (log scale) of NCK and MEK inhibitors (Trametinib, Selumetinib, or Binimetinib) for 6 days. **B** The logarithmic combination index (CI) value of NCK and the indicated MEK inhibitors in PDAC cells was determined using the Chou-Talalay method (http://www.combosyn.com). CI value indicates: <1 synergism; =1 additive; >1 antagonism. **C** Dose-response curves for PDAC cells treated with the indicated concentration (log scale) of MEK inhibitors ± 5 μM NCK for 6 days using total cell number assays. The arrow indicates fold reduction in the IC_50_ of respective MEK inhibitors in the presence of NCK.

Given that the high-throughput drug screening demonstrated a pronounced synergistic effect between NCK and Trametinib in AsPC-1 and BxPC-3 cells, the pharmacological inhibition of pBADS99 by NCK in combination with three MEK inhibitors Trametinib, Selumetinib, and Binimetinib (detailed information tabulated in SI 14) were further evaluated in KRAS^G12D^- and KRAS^G12V^-mutant PDAC by total cell number assay (Fig. 4A). In all six KRAS-mutant PDAC cell lines, NCK exhibited synergistic combination with MEK inhibitors, as demonstrated by CI < 1 by Chou-Talalay methodology [41] (Fig. 4B). Additionally, combination treatment of NCK (5 μM) - MEK inhibitors significantly increased the efficacy of MEK inhibitors compared to MEK inhibitor treatment alone in SW1990 and Panc-1 cells, as demonstrated by dose-response analysis (Fig. 4C). Notably, NCK significantly reduced the IC_50_ of Trametinib (~7-fold), Selumetinib (~37-fold), and Binimetinib (~460-fold) compared to their respective treatment alone in SW1990 cells. Furthermore, combined treatment of Panc-1 cells with NCK and MEK inhibitors resulted in an ~6-fold decrease in IC_50_ of Trametinib, ~13-fold decrease in IC_50_ of Selumetinib, and >9000-fold decrease in IC_50_ of Binimetinib compared to their single treatment in Panc-1 cells, respectively. Hence, it was demonstrated that NCK synergizes with MEK inhibitors in decreasing the survival of PDAC cells.

### MEK inhibition in KRAS^G12D^-mutant PDAC cells increases the pBADS99/BAD ratio

**Fig. 5:**
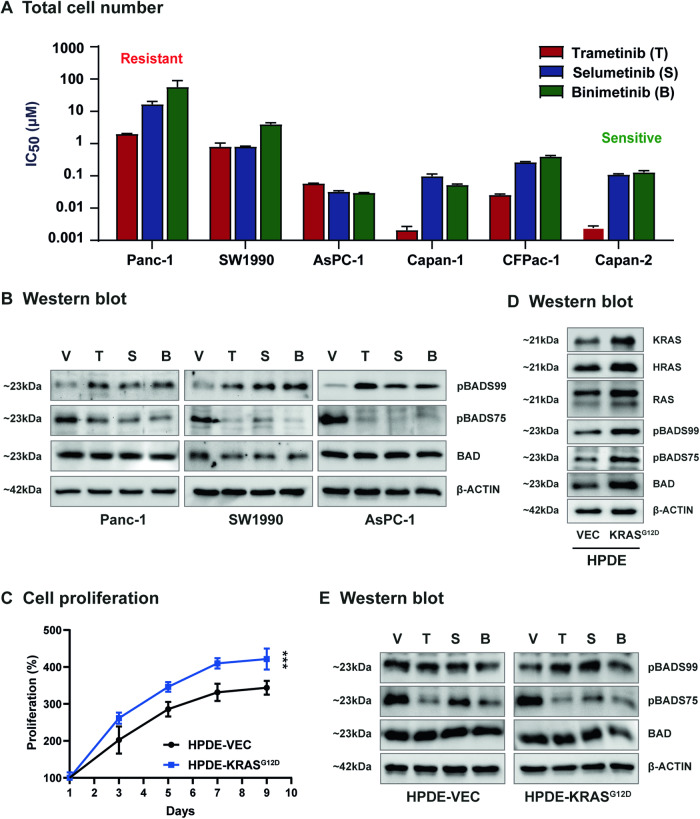
MEK inhibition causes an increase in the pBADS99/BAD ratio in KRAS-mutant PDAC cells. **A** Total cell number assay was performed to measure the IC_50_ of MEK inhibitors Trametinib (T), Selumetinib (S), or Binimetinib (B) in PDAC cells. The trypan blue exclusion method was used to determine the number of viable cells present after respective treatment for 6 days. **B** Western blot analysis was used to assess the level of BAD phosphorylation at S99 and S75 in PDAC cells after treatment with vehicle (V) and MEK inhibitors Trametinib (T), Selumetinib (S) or Binimetinib (B) for 72 h. β-ACTIN was used as input control. The sizes of detected protein bands in kDa are shown on the left. **C** Cell viability assay using AlamarBlue reagent was performed to determine the effect of forced expression of KRAS^G12D^ on cell viability in HPDE cells. Data represent means (*n* = 3). *P < 0.05, **P < 0.01, and ***P < 0.001. **D** Western blot analysis was performed to determine the expression of RAS- and BAD-related proteins in HPDE-vector (HPDE-VEC) and HPDE KRAS^G12D^ (HPDE-KRAS^G12D^) cells. β-ACTIN was used as input control. The sizes of detected protein bands in kDa are shown on the left. **E**. Western blot analysis was used to assess the level of BAD phosphorylation at S99 and S75 in HPDE-vector (HPDE-VEC) and HPDE KRAS^G12D^ (HPDE-KRAS^G12D^) cells after treatment with vehicle (V) and MEK inhibitors Trametinib (T), Selumetinib (S) or Binimetinib (B) for 72 h. β-ACTIN was used as input control. The sizes of detected protein bands in kDa are shown on the left.

Heterogenous responses of KRAS-mutant cancers towards MEK inhibitors in the clinic has been widely reported [16, 17, 42]. Herein, the effect of MEK inhibitors on cell survival and levels of BAD phosphorylation in KRAS-mutant PDAC were examined. A differential effect of MEK inhibitors on KRAS-mutant PDAC cell survival was observed, with IC_50_ ranging from 0.001 μM to 100 μM. Notably, Panc-1 and SW1990, two cell lines harboring KRAS^G12D^ activation with higher pBADS99/BAD ratios (Fig. 2A) were relatively more resistant to MEK inhibitors, Trametinib (Panc-1 IC_50_ = 1.98 μM; SW1990 IC_50_ = 0.80 μM), Selumetinib (Panc-1 IC_50_ = 16.48 μM; SW1990 IC_50_ = 0.81 μM) and Binimetinib (Panc-1 IC_50_ = 56.60 μM; SW1990 IC_50_ = 3.98 μM) compared to other KRAS-mutant PDAC cell lines (IC_50_ < 0.50 μM) (Fig. 5A). Subsequently, by western blot analysis, it was demonstrated that despite MEK inhibitors (Trametinib, Selumetinib or Binimetinib) significantly reducing pBADS75/BAD levels in KRAS^G12D^-mutant cell lines, the treatments significantly increased the levels of pBADS99/BAD (Fig. 5B, SI 15A). This observation has not been reported previously; however, the PI3K/AKT pathway is critical for the therapeutic response toward MEK inhibitors in KRAS-mutant cancer [32, 43]. Hence, the increase in the pBADS99/BAD ratio might suggest a novel mechanism contributing to the therapeutic failure of MEK inhibitors.

To further verify the finding, we transfected the immortalized normal human pancreatic duct epithelial cell line HPDE6-C7 (HPDE) with an empty *vector* or KRAS^G12D^ expression *vector* (Fig. 5C). AlamarBlue assay demonstrated a higher growth rate of the KRAS^G12D^ transfected cells compared to *vector*-transfected HPDE cells (Fig. 5C) [44, 45]. Additionally, KRAS^G12D^ transfected HPDE cells exhibited higher KRAS, HRAS, pan-RAS, pBADS99/BAD, and pBADS75/BAD levels compared to empty *vector*-transfected cells, as demonstrated by western blot analysis (Fig. 5D, SI 15B). Consistent with KRAS^G12D^-mutant PDAC cell lines, it was demonstrated that MEK inhibitor treatment produced an increase in the pBADS99/BAD ratio in KRAS^G12D^ transfected HPDE cells, but not in HPDE-*vector* cells, and reduced pBADS75/BAD levels (Fig. 5E, SI 15C). Therefore, the combination targeting of pBADS99 and MEK represents a rational strategy to potentiate the effect of MEK inhibitors in KRAS-mutant PDAC.

### NCK synergizes with MEK inhibitors in KRAS-mutant PDAC cells by promoting intrinsic apoptosis

**Fig. 6:**
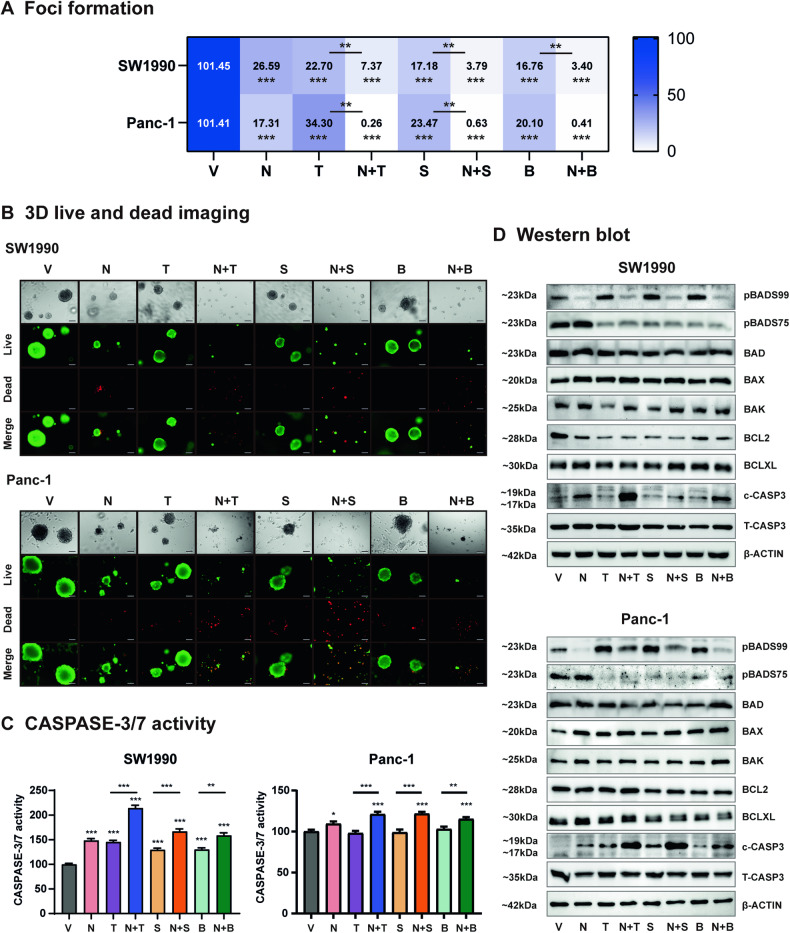
NCK synergizes with MEK inhibitors in KRAS-mutant PDAC cells by stimulating apoptosis. **A** Heatmap plot depicts quantification of crystal violet staining of foci of SW1990 and Panc-1 cells after exposure to vehicle (V), NCK (N), MEK inhibitors (T, S or B), or NCK + MEK inhibitors (N + T, N + S or N + B) for 9 days. Data represent ratio of cell viability relative to the vehicle (mean, *n* = 3). *P < 0.05, **P < 0.01, and ***P < 0.001. **B** Microscopic visualization of Calcein-AM (green) stained colonies (live) and BOBO-3 Iodide (red) stained cell debris (dead) generated by SW1990 and Panc-1 cells cultured in 3D Matrigel after exposure to vehicle (V), NCK (N), MEK inhibitors (T, S or B), or NCK + MEK inhibitors (N + T, N + S or N + B) for 12 days. Scale bars, 100 μm. **C** CASPASE 3/7 activities were evaluated in SW1990 and Panc-1 cells after the respective treatments for 72 h using the Biovision Caspase 3/7 DEVD Assay Kit. Data represent means ± SD (*n* = 3). *P < 0.05, **P < 0.01, and ***P < 0.001. **D**. Western blot analysis was used to assess the level of BAD and apoptotic proteins in SW1990 and Panc-1 after treatment with vehicle (V), NCK (N), MEK inhibitors (T, S or B), and their combinations (N + T, N + S or N + B) for 72 h. β-ACTIN was used as input control. The sizes of detected protein bands in kDa are shown on the left.

Next, the effect of combined NCK-MEK inhibitor treatment on foci-forming capacity and 3D growth of SW1990 and Panc-1 cells was evaluated. NCK and MEK inhibitors significantly inhibited the foci formation of PDAC cells, whereas combined NCK-MEK inhibition further attenuated the capacity for foci formation of SW1990 and Panc-1 cells as compared to single MEK inhibitor treatment (Fig. 6A, SI 16A). Consistently, NCK and MEK inhibitor combinations significantly reduced cell viability in 3D Matrigel compared to vehicle (Fig. 6B). The combined treatment of NCK and MEK inhibitors synergistically reduced 3D Matrigel growth of both SW1990 and Panc-1 PDAC cells, as demonstrated by an increase in the dead cell proportion (red fluorescence) and a decrease in the live cell proportion (green fluorescence) when compared to MEK inhibitor treatment alone (Fig. 6B). CASPASE 3 and 7 are effector enzymes that initiate apoptosis upon cleavage [46]. Therefore, CASPASE 3/7 activity assays were performed to evaluate the effects of combined NCK-MEK inhibition on apoptotic cell death (Fig. 6C). The treatment of SW1990 cells with either a single agent or combined NCK-MEK inhibitor resulted in a significant increase in CASPASE 3/7 activity. Combined NCK-MEK inhibition synergistically augmented CASPASE 3/7 activity compared to SW1990 cells treated with either NCK or MEK inhibitors alone (Fig. 6C). Similar directional changes in CASPASE 3/7 activity after treatment with combined NCK-MEK inhibitors were observed in Panc-1 cells (Fig. 6C).

Subsequently, the mechanistic basis underlying synergistic effects of NCK and MEK inhibitors was further analyzed using western blot assay. NCK significantly decreased the pBADS99/BAD ratio, without altering the level of pBADS75/BAD and BAD expression in PDAC cells, except for SW1990 cells, where an increase in the pBADS75/BAD ratio was also observed. Consistent with Fig. 5B, MEK inhibitors significantly decreased the pBADS75/BAD level but increased the pBADS99/BAD ratio in both PDAC cell lines (Fig. 6D, SI 16B-C). Combined NCK-MEK inhibitor treatment significantly reduced the pBADS99/BAD level augmented by MEK inhibitor treatment in PDAC cells (Fig. 6D, SI 16B-C). NCK or MEK inhibitors significantly increased the ratio of BAX/BCL-2 and BAK/BCL-2 in SW1990 cells (Fig. 6D, SI 16B). Combined NCK-Trametinib treatment significantly increased the BAK/BCL-2 ratio, whereas combined treatment of NCK-Selumetinib or Binimetinib significantly increased the BAX/BCL-2 and BAK/BCL-2 ratios in SW1990 cells. In Panc-1 cells, NCK or MEK inhibitor single treatment significantly increased the BAX/BCL-2 and BAX/BCL-XL ratio as compared to vehicle. The BAX/BCL-XL ratio was further enhanced by the combination treatments as compared to their respective MEK inhibitor (Fig. 6D, SI 16C). Combined NCK-Binimetinib also significantly increased the BAX/BCL-2 and BAK/BCL-XL ratios as compared to Binimetinib alone in Panc-1 cells. Consistent with CASPASE 3/7 activity assays (Fig. 6C), NCK significantly increased the cleaved-CASPASE3/total-CASPASE3 ratio compared to vehicle treatment. The combination of NCK-MEK inhibition also significantly increased the cleaved-CASPASE3/total-CASPASE3 ratio compared to the treatment with the respective MEK inhibitor in PDAC cells (Fig. 6D, SI 16B-C). Therefore, NCK synergizes with MEK inhibitors in KRAS-mutant PDAC cells by promoting intrinsic apoptosis.

### Combined NCK-Trametinib treatment suppresses the growth of KRAS^G12D^-mutant PDAC xenografts

**Fig. 7:**
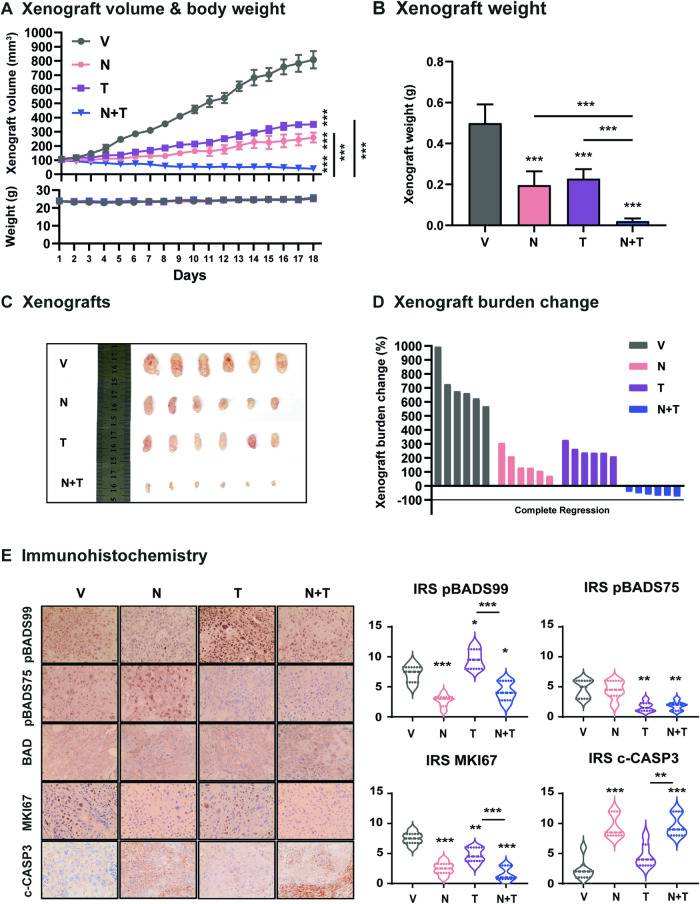
Combined NCK-Trametinib treatment suppresses the growth of KRAS^G12D^-mutant PDAC SW1990 xenografts. **A** Xenograft volume (mm^3^) of each treatment group (Vehicle (V), NCK (N), Trametinib (T) or NCK+Trametinib (N + T)) was measured daily and calculated by using the formula: 0.52 × length × [width]^2^. The animal weight of each treatment group was indicated. Data represent means ± SD (*n* = 6). *P < 0.05, **P < 0.01, and ***P < 0.001. **B** Mean xenograft weight of each treatment group after sacrifice at the end of the 18th day. Data represent means ± SD (n = 6). *P < 0.05, **P < 0.01, and ***P < 0.001. **C** Resected xenograft tumors from each treatment group (Vehicle (V), NCK (N), Trametinib (T), or NCK+Trametinib (N + T)) were shown. **D** Xenograft burden change of each treatment group (Vehicle (V), NCK (N), Trametinib (T), or NCK+Trametinib (N + T)) measured at the end of the experiment. **E**. Histological analyses and IRS scoring of pBAD at S99 and S75, MKI67, and cleaved-CASPASE3 (c-CASP3) in xenografts. Representative micrographs were taken at 200× magnification. Scale bar, 20 µm. The IRS scoring method is described in the materials & methods. Data represent means ± SD. *P < 0.05, **P < 0.01, and ***P < 0.001.

PDAC xenografts were generated by subcutaneously injecting SW1990 cells (5 ×10^6^) into male BALB-c/nude mice aged approximately 8 weeks. Upon the xenograft reaching ~100 mm^3^, the SW1990 xenograft-bearing mice were randomly grouped (*n* = 6) using a random number table method and were administered intraperitoneally (i.p.) with the vehicle, NCK (20 mg/kg *q.d*.), Trametinib (1 mg/kg *q.o.d*) or a NCK+Trametinib combination (N + T). The xenograft volume and host animal body weight were measured daily as presented in Fig. 7A. All mice were sacrificed 18 days after the commencement of the drug treatment as the xenograft volume of the vehicle-treated mice reached approximately 800-1000 mm^3^. On the fifth day of drug treatment, significant reductions in xenograft volume were observed in the single agent (NCK or Trametinib) and combined N + T treated groups compared to the vehicle-treated mice (Fig. 7A, SI 17A). Starting from the eighth day of drug treatment, the xenografts of mice receiving combined N + T treatment were significantly smaller in terms of volume compared to the xenograft of the NCK or Trametinib group (Fig. 7A, SI 17A). Indeed, all combination-treated xenografts regressed below the initial volume at the commencement of drug treatment. Consistent results were observed with xenograft weight (Fig. 7B) and resected xenografts (Fig. 7C).

Furthermore, xenograft burden change (Fig. 7D) was demonstrated by waterfall plots and modified response evaluation criteria in solid cancers (mRECIST) [47] was utilized to categorize the drug response into progressive disease (PD), stable disease (SD), partial response (PR), or complete response (CR) as per the best response and the best average response (Table 4). 6/6 (100%) of the vehicle-treated xenografts, 4/6 (66.7%) of the NCK-treated xenografts and 5/6 (83.3%) of the Trametinib-treated xenografts were categorized as PD. 2/6 (33.3%) of the NCK-treated xenografts and 1/6 (16.7%) of the Trametinib-treated xenografts were categorized as SD. Synergistically, the combination treatment of NCK and Trametinib caused 2/6 (33.3%) SD and 4/6 (66.7%) PR in the xenografts (Table 4). No significant change in body weight (Fig. 7A) nor in the morphology and relative weight of vital organs (SI 18-19) were observed, suggesting tolerability of the drug treatments.

**Table 4:** mRECIST evaluation of drug response (V, N, T and N + T).

mRECIST	V	N	T	N + T
mCR (%)	0	0	0	0
mPR (%)	0	0	0	66.7
mSD (%)	0	33.3	16.7	33.3
mPD (%)	100	66.7	83.3	0

Next, histological analyses were performed on the resected xenograft specimens. Consistent with in vitro observations (Fig. 6D), xenograft specimens resected from NCK-treated mice exhibited a significant reduction in pBADS99 levels and the pBADS99/BAD ratio but no change in the pBADS75 levels nor the ratio of pBADS75/BAD compared to xenograft specimens of the vehicle-treated group (Fig. 7E, SI 17B-C). However, Trametinib inhibited pBADS75 levels and the pBADS75/BAD ratio but increased the pBADS99 levels and the pBADS99/BAD ratio in the xenograft specimens compared to vehicle treatment. The combined N + T treatment significantly attenuated the increased pBADS99 levels and the pBADS99/BAD ratio in the xenograft specimens induced by Trametinib. The xenograft specimens of the combination treatment also exhibited lower pBADS75 levels and a lower pBADS75/BAD ratio compared to the vehicle, due to the effect of Trametinib. None of the treatments altered the level of BAD expression in the xenografts, as demonstrated by IRS analysis (Fig. 7E, SI 17D). Subsequently, the effect of treatments on MKI67, a cell proliferation marker, was examined in the resected xenograft specimens. Compared with vehicle-treated mice, xenograft specimens of all treated groups (NCK, Trametinib, or N + T treatment) exhibited significantly lower MKI67 labeling. The combined N + T treatment further reduced the IRS of MKI67 in the xenograft specimens compared to Trametinib treatment alone (Fig. 7E). Consistent with in vitro observations (Fig. 6C), NCK treatment significantly increased cleaved-CASPASE 3 levels compared to vehicle. Additionally, the xenografts of N + T-treated mice exhibited a significant increase in cleaved-CASPASE 3 levels compared to xenografts of the Trametinib-treated group (Fig. 7E). Hence, combination treatment of NCK and Trametinib suppresses the xenograft growth of KRAS^G12D^-mutant PDAC.

## Discussion

Lack of specific and accessible early-stage biomarkers, therapy resistance, and consequent recurrence of PDAC demand novel and potent strategies to improve patient survival [48, 49]. As the most frequently mutated *RAS* gene (84%) in human cancer [9, 50], KRAS mutations are associated with a worse prognosis in PDAC (90%), colorectal cancer (50%), non-small cell lung cancer (30%) and other human malignancies [6]. Indeed, PDAC patients with wild-type KRAS generally exhibited better prognosis than those harboring mutant KRAS [51], even though patients with wild-type KRAS PDAC frequently harbor activating BRAF mutations, thus similarly generating constitutive activation of the p44/42 MAPK pathway [52]. KRAS^G12D^, the predominant KRAS mutant subtype in PDAC is reported to activate both PI3K/AKT and p44/42 MAPK signaling cascades, whereas KRAS^G12V^ or KRAS^G12C^ predominantly activate Ral signaling [53]. Despite the activation of the p44/42 MAPK pathway by mutant KRAS, compensatory feedback activation has been reported to be associated with failure of MEK inhibitors as a monotherapy in clinical trials for the treatment of PDAC and other cancers harboring KRAS mutation [32, 54–56]. Additionally, clinical trials combining MEK inhibitors and gemcitabine have failed to demonstrate significant improvements in PDAC patient survival compared with gemcitabine alone [57, 58]. In fact, substantial research has reported an increase in PI3K pathway activity following the inhibition of the p44/42 MAPK pathway in KRAS-mutant cancers [20, 30, 31]. The activation of PI3K/AKT as an escape mechanism to vertical suppression of the EGFR/RAS/MAPK pathway by EGFR and MEK inhibitors in KRAS-mutant colorectal cancer has also been reported and is linked to drug resistance and disease recurrence [31]. Since dual inhibition of both p44/42 MAPK and PI3K/AKT pathways has been shown to potentially overcome resistance, and achieve optimal cellular and xenograft growth inhibition [32, 33, 56, 59], the therapeutic potential of concurrently targeting these critical effector pathways has been widely explored. Indeed, various clinical trials have been executed to explore the efficacy of combining MEK inhibitors with PI3K/AKT pathway inhibition in PDAC by using a pan-PI3K inhibitor (NCT01155453; NCT01363232) [60], a pan-AKT inhibitor (NCT01021748; NCT01658943) [61], a mTOR inhibitor (NCT00955773) [62] or a dual PI3K and mTOR inhibitor (NCT01337765) [63]. Combined targeting of mTORC1/2, downstream of AKT, with MEK or KRAS^G12C^ inhibitors has been reported to be synergistic in promoting cell death and inhibiting xenograft growth in KRAS-mutant PDAC [32]. However, toxicity associated with simultaneously blocking these two critical effector pathways has greatly limited their clinical potential [64]. Therefore, identifying well-tolerated synergistic therapeutic strategies are vital to ameliorate patient outcomes for this aggressive cancer. Given that BAD is a vital downstream mediator of RAS effector pathways mediating cancer cell survival and apoptosis [25], targeting its phosphorylation may be and is demonstrated herein, to be advantageous for the treatment of KRAS-mutant PDAC. In addition to KRAS, loss-of-function mutation of TP53 and SMAD4 homozygous deletion (HD) in the majority of the PDAC patients may potentially increase the therapeutic vulnerability to pBAD inhibition [65], due to the interaction of TP53 with BAD [66, 67] and in inducing PTEN expression [68], and loss of SMAD4 with PI3K/AKT pathway activation [69, 70].

In this study, for the first time, BAD phosphorylation was demonstrated as a critical modulator of MEK inhibitor sensitivity in PDAC; and uncovered the potential capacity of dual targeting pBADS99-MEK for KRAS-mutant PDAC treatment. Since it was demonstrated that NCK possesses high oral bioavailability and a therapeutic window in vivo, its synergistic combination with a MEK inhibitor may overcome toxicity issues associated with more upstream concurrent inhibition of the p44/42 MAPK and PI3K/AKT pathways reported earlier [64]. Herein, it was further demonstrated that the synergy observed in vitro provoked significant regression of the KRAS-mutant PDAC xenografts at well-tolerated doses. Even though a complete response was not observed at the end of the treatment, as xenografts of the vehicle group reached humane endpoint, given the efficacy observed it is believed that the prolongation of the treatment time, optimization of dosage or supplementation with a third drug will markedly enhance the therapeutic response and thus warrants future investigation. In addition to MEK inhibition, high-throughput drug screening assays demonstrated that pBADS99 inhibition by NCK synergizes with most compounds targeting receptor tyrosine kinases (RTKs) or JAK/STAT in KRAS^G12D^-mutant and wild-type PDAC cells. Given that RTKs mediates the activation of PI3K/AKT and p44/42 MAPK signaling, two RAS effector pathways upstream of BAD phosphorylation [71], it was thus reasoned that the synergistic combinations with NCK were observed possibly by preventing feedback mechanisms within the two pathways. In fact, in a recent study exploring synergistic combinatorial therapeutic strategies for TNBC, the marked efficacy of combined targeting of pBADS99 and RTKs specifically VEGFR or c-MET was reported [72]. Consistently, herein, the c-MET & ALK inhibitor, Crizotinib, was observed to synergize with NCK to reduce the viability of PDAC cells. As for JAK/STAT signaling, JAK or PIM inhibitors were found to synergize with NCK in the combinatorial drug screening assay. PIM-1 and PIM-2, downstream of JAK/STAT are pro-survival kinases of BAD promoting its phosphorylation at murine S112 (human S75) [73, 74]. Co-inhibition of PIM1 and ERK or inhibition of JAK2 leads to reduced cell survival by dephosphorylation of murine BAD at S112 [75]. However, due to the hyperactivation of RAS effector pathways (PI3K/AKT and p44/42 MAPK) in RAS-mutant cells, JAK2 inhibition alone was reported to be insufficient to dephosphorylate BAD, thereby associated with the failure of JAK inhibitor monotherapy [75]. Therefore, it was suggested that co-inhibition of RAS effector pathways and JAK yields a superior therapeutic response than JAK inhibition alone [75]. Hence, the efficacy of combination of pBADS99 inhibition with JAK/STAT inhibition might be promising and worth further preclinical investigation.

Collectively, the results herein provide a mechanism-based preclinical and translational rationale, and support a distinct therapeutic opportunity in targeting BAD-mediated survival; and concurrently inhibiting pBADS99 and MEK in KRAS-mutant cancers. Due to the high frequency of KRAS alterations in human malignancies, these findings could be extended to potentially provide a significant clinical benefit across a broad cancer patient population with KRAS mutation. A graphical summary of the study has been included in SI 20.

## Materials and Methods

### Cell culture and reagents

SW1990, Panc-1, AsPC-1, and CFPac-1 were purchased from Procell Life Science & Technology Co. Ltd (Wuhan, China); BxPC-3, Capan-2, and HPDE6-C7 were purchased from BNBio Tech Co. Ltd; Capan-1 was purchased from ATCC. All cell lines were maintained as per the manufacturer’s propagation instructions. HPDE-KRAS^G12D^ cells were established by transfecting HPDE6-C7 cells with pCMV-KRAS^G12D^ plasmid (Miaoling Biology, Wuhan, China). CRISPR-Cas9 homology-directed repair (HDR) assay was carried out as previously described following Feng Zhang’s protocol [76] with sequence listed in Supplementary Information (SI) 21. The transfections were carried out using Lipofectamine 3000 (Thermo Fischer Scientific, Waltham, MA, USA).

### Tissue microarray

The tissue microarray (PAC1602) was obtained from Chenxue Biotech Co. Ltd (Guangzhou, China). Consent for the use of the tissue samples and clinical data was obtained by Chenxue Biotech Co. Ltd (Guangzhou, China). Immunohistochemistry (IHC) staining and scoring were performed as previously described using the antibodies tabulated in SI 22 [34]. The staining results were assessed and confirmed by two independent researchers blinded to the clinical data.

### High-throughput screening assay

High-throughput screening assay was performed in AsPC-1 or BxPC-3 cells using Cambridge Cancer Compound Library (SelleckChem, Houston, TX, USA) (SI 6-7). IC_25_ of the respective compound (predicted with Genomics of Drug Sensitivity in Cancer (https://www.cancerrxgene.org/) in combination with three log-doses of NCK (0.1, 1, and 10 μM) was utilized, and cell viability was measured after 72 h with AlamarBlue reagent and fluorescence was measured using a Tecan microplate reader.

### Oncogenic analysis and western blot analysis

Total cell number, foci formation, 3D Matrigel growth assays, live/dead analysis, cell cycle and apoptotic flow cytometry assays were performed as previously described [36, 76]. Live/Dead analysis was performed according to the manufacturer’s instructions using LIVE/DEAD™ Cell Imaging Kit (Thermo Fisher Scientific, MA, USA). CASPASE 3/7 assay (Biovision, CA, USA) was performed following the manufacturer’s protocol. Apoptotic cell populations were examined by using the Annexin V-FITC/PI apoptosis assay kit (Neobioscience, Shenzhen, China) following the manufacturer’s instruction. Cell cycle and apoptotic assays were carried out using Cytoflex Flow Cytometer (Beckman, CA USA). Combination index (CI) analysis was performed using the Chou-Talalay method. Western blot analysis was performed as previously described [77, 78] using the antibodies tabulated in SI 22. All functional assays were performed in a medium with 2% FBS.

### Xenografts

Xenograft study was performed as previously described [79]. The xenograft study was approved by the Laboratory Animal Ethics Committee (Certificate number: YW) at Peking University Shenzhen, and ethical approval was obtained from Tsinghua Shenzhen International Graduate School (Number: 9, Year 2020). Drug responses were analyzed by Modifying Response Evaluation Criteria in Solid Tumors (mRECIST) [47, 80]. IHC analysis of xenograft histology sections for pBADS99, pBADS75, BAD, MKI67, and cleaved CASPASE-3 were analyzed as previously described [79] using antibodies tabulated in SI 22.

### Statistical analysis

All experiments in the study were carried out at least 3 times (in vitro) or 6 times (in vivo) and displayed as Mean ± SD. The software package Prism5 (GraphPad Software, Inc., CA, USA) was utilized for statistical analysis. Results were analyzed by two-tailed unpaired *student’s t-test* and ANOVA analysis when two-group and multiple samples were compared, respectively. The significant levels in all statistical analyses were set at *P < 0.05, **P < 0.01, and ***P < 0.001

### Supplementary information

Supplementary File

Supplementary File-Original Western Blot

Checklist

## Data Availability

Original western blots are available in the supplementary file. Other data sets used in this study are available from the corresponding author on reasonable request.
