# Supplementary material for: Concurrent inhibition of pBADS99 synergistically improves MEK inhibitor efficacy in KRASG12D-mutant pancreatic ductal adenocarcinoma
Source: Cell Death Dis. 2024 Feb 26;15(2):173. doi: 10.1038/s41419-024-06551-7 (PMC10897366; doi:10.1038/s41419-024-06551-7)
Supplement: Supplementary file 1 — Supplementary File [file 41419_2024_6551_MOESM1_ESM.pdf]

Supplementary Information for

Concurrent inhibition of pBADS99 synergistically improves MEK inhibitor efficacy in KRAS^{G12D}-mutant pancreatic ductal adenocarcinoma

Yan Qin Tan[1,2,*], Bowen Sun[1,*], Xi Zhang[1,3], Shuwei Zhang[1], Hui Guo[1], Basappa Basappa[4], Tao Zhu[3,5,6], Gautam Sethi[7], Peter E. Lobie[1,3,#] and Vijay Pandey[1,#]

[1]Institute of Biopharmaceutical and Health Engineering and Tsinghua Berkeley Shenzhen Institute, Tsinghua Shenzhen International Graduate School, Tsinghua University, Shenzhen 518055, People's Republic of China.

[2]Food Science and Technology Program, Department of Life Sciences, BNU-HKBU United International College, Zhuhai 519087, Guangdong, People's Republic of China.

[3]Shenzhen Bay Laboratory, Shenzhen 518055, Guangdong, People's Republic of China.

[4]Laboratory of Chemical Biology, Department of Studies in Organic Chemistry, University of Mysore, Manasagangotri, 570006 Mysore, India.

[5]Department of Oncology, The First Affiliated Hospital of USTC, Center for Advanced Interdisciplinary Science and Biomedicine of IHM, Division of Life Sciences and Medicine, University of Science and Technology of China, Hefei, Anhui 230027, People's Republic of China.

[6]Hefei National Laboratory for Physical Sciences, University of Science and Technology of China, Hefei, Anhui 230027, People's Republic of China.

[7]Department of Pharmacology, Yong Loo Lin School of Medicine, National University of Singapore, Singapore 117600, Singapore; NUS Centre for Cancer Research, Yong Loo Lin School of Medicine, National University of Singapore, Singapore 117599, Singapore.

[*] These authors contributed equally to this work.

[#]Correspondence to:
Peter E. Lobie, email: pelobie@sz.tsinghua.edu.cn
Vijay Pandey, email: vijay.pandey@sz.tsinghua.edu.cn

Supplementary Information 1

Cohort	Total (N)	High (%)	Low (%)	P-value
**Gender**				**0.089**
Male	24	42	58	
Female	31	55	45	
**Age**				**<0.001*** **
<60	21	33	67	
>=60	34	59	41	
**Diameter**				**0.396**
<=4	33	52	48	
>4	22	45	55	
**Grade**				**<0.001*** **
1	6	83	17	
2	16	38	63	
3	33	48	52	
**TNM**				**0.037***
I	24	46	54	
II	15	60	40	
III	6	50	50	
IV	10	40	60	
**Lymph node metastasis**				**>0.999**
Yes	18	50	50	
No	37	49	51	
**Distant metastasis**				**0.010** **
Yes	9	33	67	
No	46	52	48	

Correlation analysis between pBADS75/BAD level and clinicopathological features of PDAC patient. *$P < 0.05$, **$P < 0.01$, and ***$P < 0.001$. The immunoreactive score (IRS) ratio ≥ 2 was categorized as high pBADS75/BAD and IRS ratio < 2 was categorized as low pBADS75/BAD in PDAC patient cohort.

Supplementary Information 2

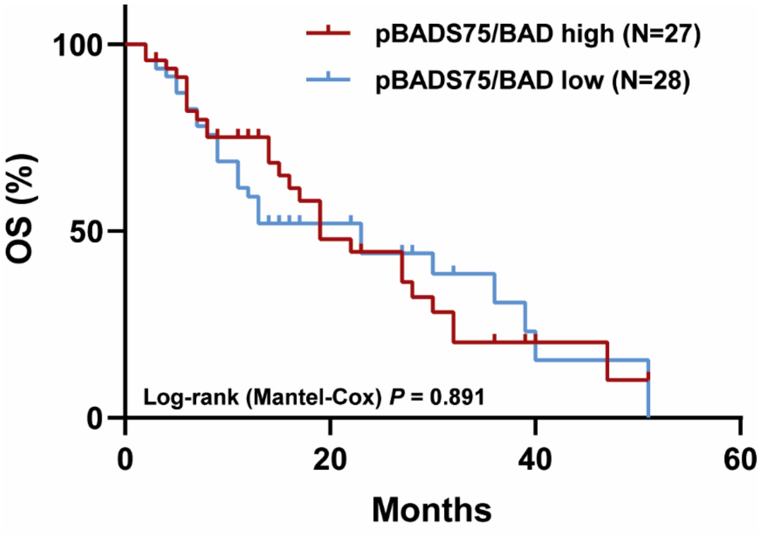

Kaplan–Meier analysis of overall survival in PDAC stratified according to pBADS75/BAD low or high expression in PDAC tissues.

Supplementary Information 3

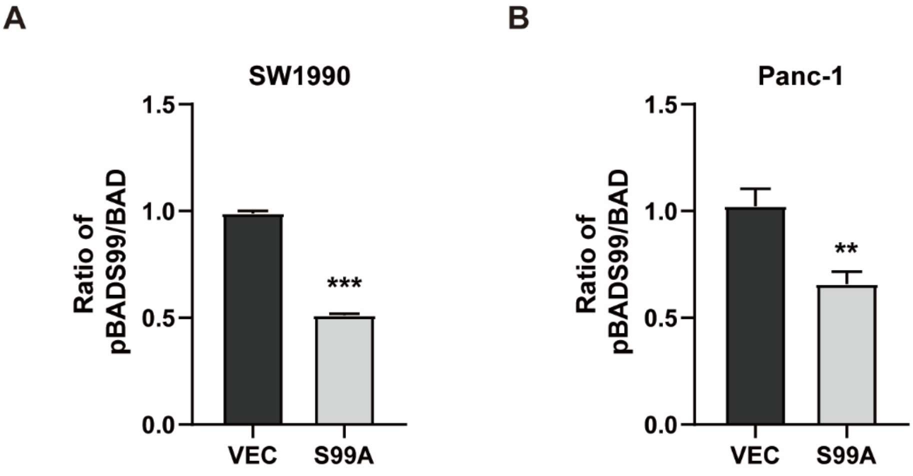

A. Densitometric analysis of western blots in Figure 1B. SW1990 cells were transfected with vector or S99A HDR plasmid. Densitometric analysis of protein blots was determined using ImageJ software (https://imagej.nih.gov/ij/). Statistical changes were assessed by using ANOVA. $*P < 0.05$, $**P < 0.01$, and $***P < 0.001$.

B. Densitometric analysis of western blots in Figure 1C. Panc-1 cells were transfected with vector or S99A HDR plasmid. Densitometric analysis of protein blots was determined using ImageJ software (https://imagej.nih.gov/ij/). Statistical changes were assessed by using ANOVA. $*P < 0.05$, $**P < 0.01$, and $***P < 0.001$.

Supplementary Information 4

Cell Line	KRAS Mutational Status			Cancer Pathology				
	Status	Site	Mutation	Type	Age	Gender	Ethnicity	Derivation
SW1990	**MUT**	Codon 12	G12D (ASP)	PDAC	56	Male	CAU	Spleen metastasis
Panc-1	**MUT**	Codon 12	G12D (ASP)	PDAC	56	Male	CAU	Primary
AsPC-1	**MUT**	Codon 12	G12D (ASP)	PDAC	62	Female	CAU	Ascites
CFPAC-1	**MUT**	Codon 12	G12V (VAL)	PDAC	26	Male	CAU	Liver metastasis
Capan-1	**MUT**	Codon 12	G12V (VAL)	PDAC	40	Male	CAU	Liver metastasis
Capan-2	**MUT**	Codon 12	G12V (VAL)	PDAC	56	Male	CAU	Primary

Detailed information on KRAS mutational status and cancer pathology of KRAS mutant PDAC cell lines. Abbreviations: MUT, Mutation; ASP, Aspartic acid; VAL, Valine; CAU, Caucasian

Supplementary Information 5

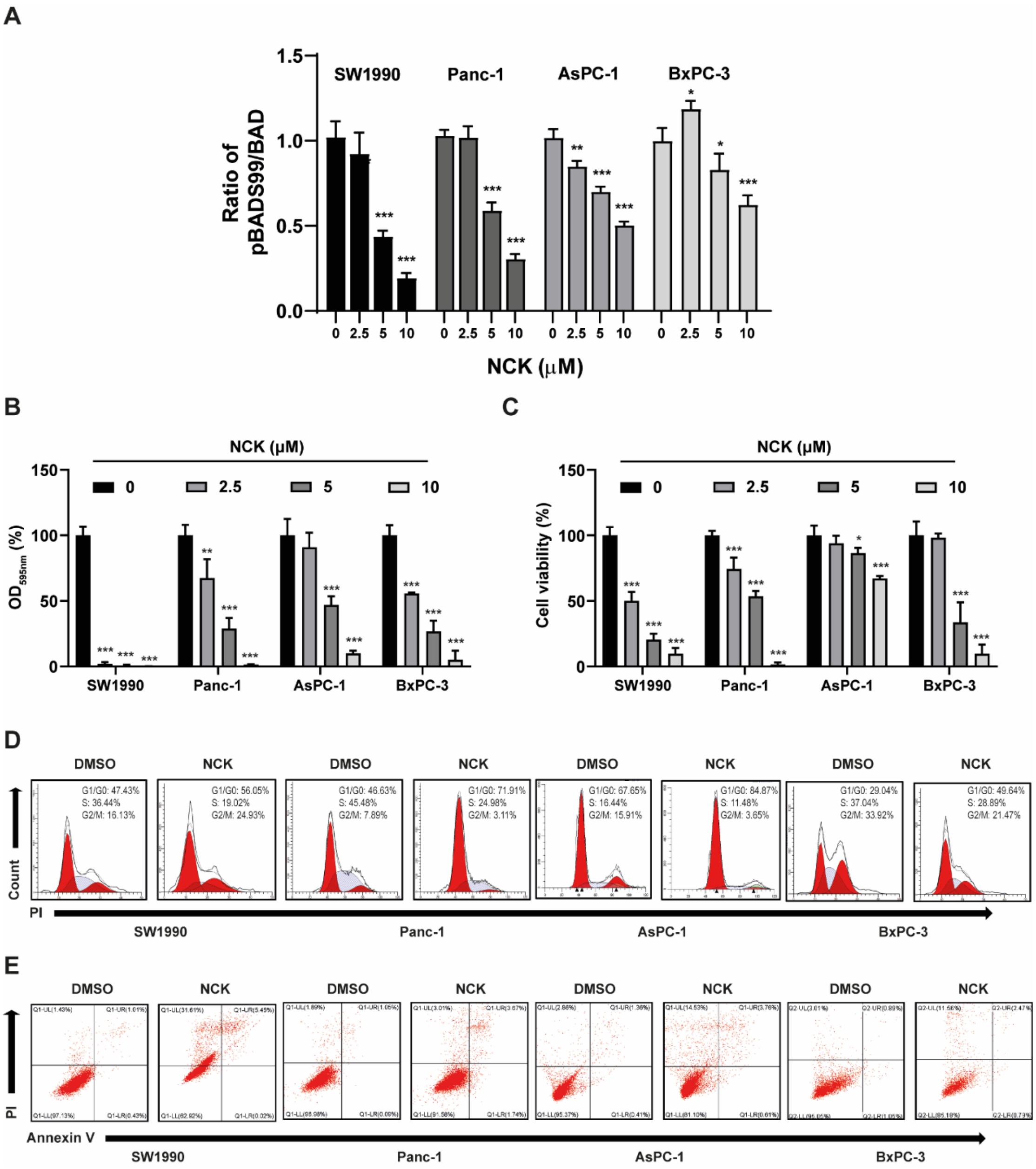

A. Densitometric analysis of western blots in Figure 2A. PDAC cells were treated with 0-10 μM of NCK. Densitometric analysis of protein blots was determined using ImageJ software (https://imagej.nih.gov/ij/). Statistical changes were assessed by using ANOVA. **P* < 0.05, ***P* < 0.01, and ****P* < 0.001.

B. Crystal violet staining of foci in colonies generated by PDAC cells after exposure to 0-10 μM of NCK. Cell viability was measured by eluting the crystal violet with methanol and detect absorbance at 595nm using microplate reader (Tecan Spark®, Switzerland). Data represent means ± SD (n=3). **P* < 0.05, ***P* < 0.01, and ****P* < 0.001.

C. Cell viability of colonies generated by PDAC cells in 3D Matrigel after exposure to 0-10 μM of NCK were determined using the AlamarBlue viability assay. Data represent means ± SD (n=3). *P < 0.05, **P < 0.01, and ***P < 0.001.

D. Representative flow cytometry plots using PI staining for DNA of PDAC cells measured after treatment with 10 μM NCK using flow cytometry analysis at 72 hours as described in materials and methods.

E. Representative flow cytometry plots of Annexin-V and propidium iodide (PI) stained apoptotic cell death of PDAC cells measured after treatment with 10 μM NCK using flow cytometry analysis. Annexin V-FITC staining is indicated on the x axis, and PI staining is indicated on the y axis. The lower left quadrants represent live cells, the lower right quadrants represent early apoptotic cells, the upper left quadrants represent necrotic cells, and the upper right quadrants display late apoptotic cells. Acquisition of Annexin V and PI data are presented as a percentage (%) in each quadrant. Early apoptotic cells are referred to as Annexin-V positive and late apoptotic cells are referred to as Annexin-V and PI double positive.

Supplementary Information 6

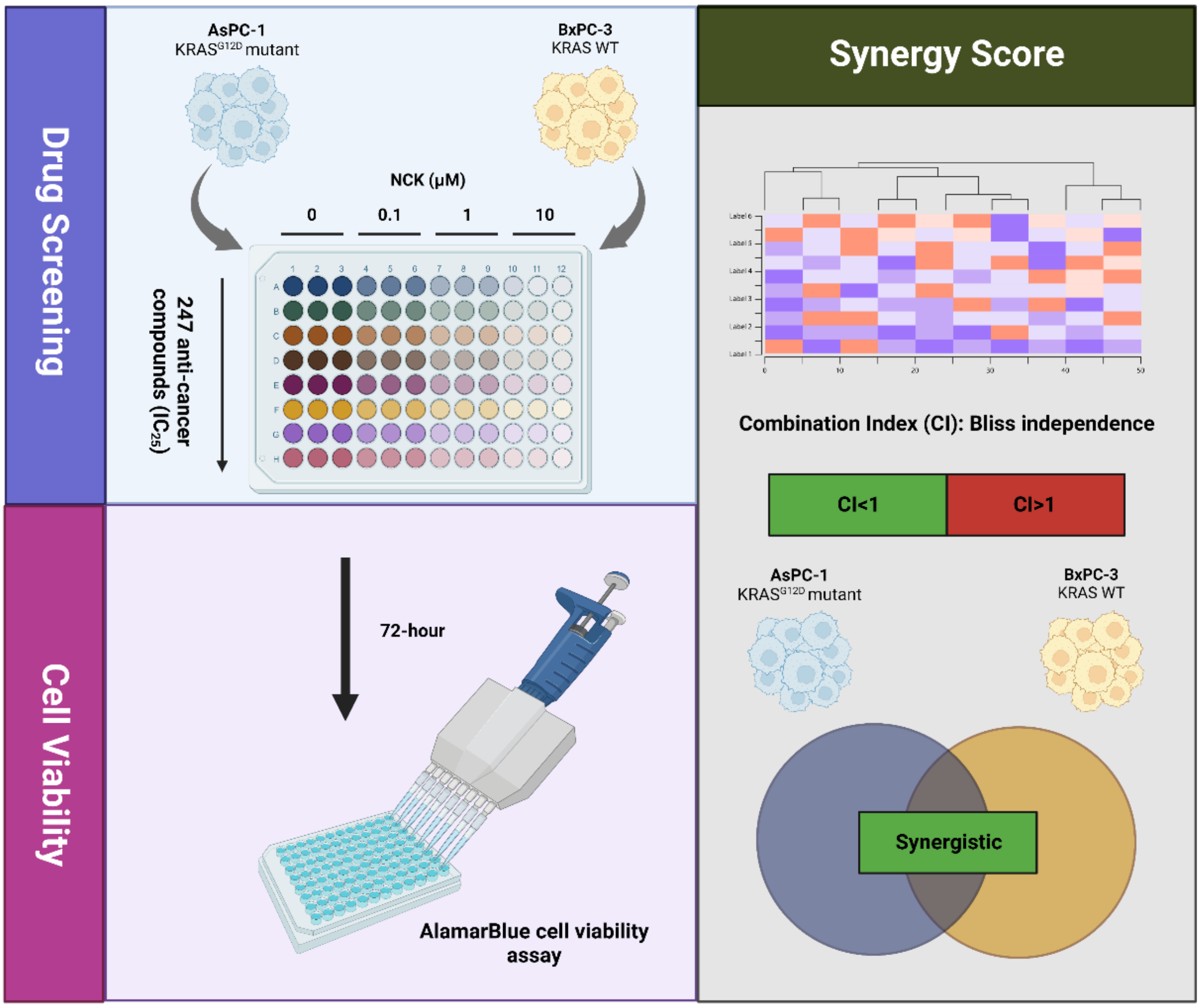

Schematic of high-throughput compound library screening assay. 247 anti-cancer agents (IC_{25}) were combined with NCK at three log doses (0.1, 1 and 10 μM) in PDAC AsPC-1 and BxPC-3 cells. Cell viability was determined 72-hour post-treatment with AlamarBlue viability assay. Created with BioRender.com.

Supplementary Information 7

Pathway	Target	Compound name
**Angiogenesis**	Aurora Kinase,FLT3,VEGFR	ENMD-2076
	Bcr-Abl	Nilotinib (AMN-107)
	Bcr-Abl	Bafetinib (INNO-406)
	Bcr-Abl	Rebastinib (DCC-2036)
	Bcr-Abl,c-Kit,Src	Dasatinib
	Bcr-Abl,DUB	Degrasyn (WP1130)
	Bcr-Abl,FGFR,PDGFR,VEGFR	Ponatinib (AP24534)
	BTK	Ibrutinib (PCI-32765)
	FAK	PF-573228
	FAK	PF-562271
	FGFR,VEGFR	PD173074
	FLT3	Quizartinib (AC220)
	HIF	2-Methoxyestradiol (2-MeOE2)
	Src	Saracatinib (AZD0530)
	Src	Bosutinib (SKI-606)
	Src	KX2-391
	Syk	R406 (free base)
	VDA	Plinabulin (NPI-2358)
**Apoptosis**	Autophagy,Bcl-2	Obatoclax Mesylate (GX15-070)
	Bcl-2	TW-37
	Bcl-2,Autophagy	ABT-737
	Caspase	PAC-1
	E3 Ligase ,Mdm2	Nutlin-3
	E3 Ligase ,p53	JNJ-26854165 (Serdemetan)
	Survivin	YM155 (Sepantronium Bromide)
	TNF-alpha	Lenalidomide (CC-5013)
	TNF-alpha	Necrostatin-1
**Cell Cycle**	APC,E3 Ligase	TAME
	Aurora Kinase	Tozasertib (VX-680, MK-0457)
	Aurora Kinase	Alisertib (MLN8237)
	Aurora Kinase	Barasertib (AZD1152-HQPA)
	Aurora Kinase	SNS-314
	Aurora Kinase	AMG-900
	Aurora Kinase,Bcr-Abl,c-RET,FGFR	Danusertib (PHA-739358)

	Aurora Kinase,CDK	JNJ-7706621
	Aurora Kinase,VEGFR	CYC116
	Autophagy,ROCK	Y-27632 2HCl
	CDK	SNS-032 (BMS-387032)
	CDK	Roscovitine (Seliciclib,CYC202)
	CDK	PHA-793887
	CDK	Flavopiridol HCl
	CDK	NU6027
	Chk	AZD7762
	Chk	Rabusertib (LY2603618)
	PLK	BI 2536
	PLK	GSK461364
	PLK	Volasertib (BI 6727)
	Rho	Azathioprine
	Rho	NSC 23766
	Wee1	Adavosertib （MK-1775）
**Cytoskeletal Signaling**	Autophagy,Microtubule Associated	Paclitaxel
	Autophagy,Microtubule Associated	Vincristine sulfate
	Autophagy,Microtubule Associated	Nocodazole
	HDAC	Abexinostat (PCI-24781)
	HDAC	Pracinostat (SB939)
	HSP (e.g. HSP90)	Elesclomol (STA-4783)
	HSP (e.g. HSP90)	Luminespib (AUY-922, NVP-AUY922)
	HSP (e.g. HSP90)	Alvespimycin (17-DMAG) HCl
	HSP (e.g. HSP90)	BIIB021
	Kinesin	Ispinesib (SB-715992)
	Kinesin	AZ 3146
	Microtubule Associated	ABT-751 (E7010)
	Microtubule Associated	Combretastatin A4
**DNA Damage**	ATM/ATR	KU-55933 (ATM Kinase Inhibitor)
	ATM/ATR	KU-60019
	DNA alkylator	Altretamine
	DNA alkylator	Cyclophosphamide Monohydrate
	DNA Methyltransferase	Azacitidine
	DNA Methyltransferase,HER2,Telomerase,EGFR,Fatty Acid Synthase	(-)-Epigallocatechin Gallate

	DNA/RNA Synthesis	Capecitabine
	DNA/RNA Synthesis	Cladribine
	DNA/RNA Synthesis	Fluorouracil (5-Fluoracil, 5-FU)
	DNA/RNA Synthesis	Clofarabine
	DNA/RNA Synthesis	Lomustine
	DNA/RNA Synthesis	Oxaliplatin
	DNA/RNA Synthesis,Autophagy	Temozolomide
	HDAC	Sodium Phenylbutyrate
	PARP	Veliparib (ABT-888)
	PARP	Olaparib (AZD2281, Ku-0059436)
	PARP	Iniparib (BSI-201)
	PARP	Rucaparib (AG-014699,PF-01367338) phosphate
	PARP	AG-14361
	Telomerase	BIBR 1532
	Topoisomerase	Doxorubicin (Adriamycin) HCl
	Topoisomerase	Etoposide
	Topoisomerase	Topotecan HCl
	Topoisomerase	Irinotecan HCl Trihydrate
	Topoisomerase	Mitoxantrone 2HCl
	Topoisomerase	Daunorubicin HCl
**Endocrinology & Hormones**	Androgen Receptor	Andarine
	Androgen Receptor	Bicalutamide
	Androgen Receptor	Enzalutamide (MDV3100)
	Androgen Receptor	Flutamide
	Androgen Receptor,Estrogen/progestogen Receptor	Megestrol Acetate
	Aromatase	Anastrozole
	Aromatase	Exemestane
	Aromatase	Letrozole
	Aromatase	Aminoglutethimide
	Aromatase	Formestane
	Estrogen/progestogen Receptor	Raloxifene HCl
	Estrogen/progestogen Receptor	Estrone
	Estrogen/progestogen Receptor	Estradiol
	Estrogen/progestogen Receptor	Toremifene Citrate
	Estrogen/progestogen Receptor	Mifepristone
	Estrogen/progestogen Receptor,Autophagy	Tamoxifen Citrate

	Glucocorticoid Receptor	Hydrocortisone
	GPR	GW9508
**Epigenetics**	Autophagy,HDAC	Vorinostat (SAHA, MK0683)
	DNA Methyltransferase	Decitabine
	DNA Methyltransferase	RG108
	EGFR,HDAC,HER2	CUDC-101
	HDAC	Entinostat (MS-275)
	HDAC	Belinostat (PXD101)
	HDAC	Mocetinostat (MGCD0103)
	Histone Acetyltransferase	MG149
	Histone Methyltransferase	BIX 01294
	Sirtuin	SRT1720 HCl
	Sirtuin	Selisistat (EX 527)
	Sirtuin	Sirtinol
**GPCR & G Protein**	Endothelin Receptor	Zibotentan (ZD4054)
	S1P Receptor	Fingolimod (FTY720) HCl
	S1P Receptor	SKI II
	SGLT	Dapagliflozin
	SGLT	Phloretin
	SGLT	Canagliflozin
**Immunology & Inflammation**	Immunology & Inflammation related	Cyclosporin A
	ROS	Febuxostat
**JAK/STAT**	JAK	Ruxolitinib (INCB018424)
	JAK	Momelotinib (CYT387)
	JAK	NVP-BSK805 2HCl
	JAK	Fedratinib (SAR302503, TG101348)
	JAK	Tofacitinib (CP-690550,Tasocitinib)
	JAK	CEP-33779
	JAK	S-Ruxolitinib (INCB018424)
	Pim	SGI-1776 free base
	Pim	SMI-4a
**MAPK**	ERK	FR 180204
	MEK	Selumetinib (AZD6244)
	MEK	PD0325901
	MEK	TAK-733
	MEK	Trametinib (GSK1120212)
	p38 MAPK	Doramapimod (BIRB 796)
	p38 MAPK	PH-797804

	PDGFR,Raf,VEGFR	Sorafenib Tosylate
	Raf	GDC-0879
	Raf	Vemurafenib (PLX4032, RG7204)
	Raf	SB590885
	Raf	AZ 628
**Metabolism**	Casein Kinase	Silmitasertib (CX-4945)
	CETP	Dalcetrapib (JTT-705, RO4607381)
	Dehydrogenase	Mycophenolate Mofetil
	Dehydrogenase	Disulfiram
	Dehydrogenase	gossypol-Acetic acid
	Dehydrogenase	Mycophenolic acid
	DHFR	Methotrexate
	FAAH	PF-3845
	HMG-CoA Reductase	Simvastatin
	HMG-CoA Reductase	Fluvastatin Sodium
	LDL	Ezetimibe
	Lipoxygenase	Zileuton
	PDE	Anagrelide HCl
	PPAR	GSK3787
	Transferase	Tipifarnib
	Transferase	Lomeguatrib
	Vitamin	Doxercalciferol
**Microbiology**	CCR	Maraviroc
**Neuronal Signaling**	COX	Celecoxib
	GABA Receptor,HDAC,Autophagy	Valproic acid sodium salt (Sodium valproate)
**NF-κB**	E2 conjugating,IκB/IKK	BAY 11-7082
	IκB/IKK	TPCA-1
	IκB/IKK	Bay 11-7085
	NF-κB	Triptolide (PG490)
	NF-κB,TNF-alpha	QNZ (EVP4593)
**Others**	Autophagy,IL Receptor	Dexamethasone (DHAP)
	FXR	Turofexorate Isopropyl (XL335)
	FXR	GW4064
	Liver X Receptor	GW3965 HCl
	Others	Lonidamine
	Others	GSK650394
	Others	SecinH3
	Substance P	Aprepitant
	Akt	MK-2206 2HCl

**PI3K/Akt/mTOR**	Akt	GSK690693
	AMPK,Fatty Acid Synthase	A-769662
	ATM/ATR,mTOR	Torin 2
	Autophagy,DNA-PK,mTOR,PI3K	PI-103
	Autophagy,mTOR	Rapamycin (Sirolimus)
	Autophagy,PI3K	LY294002
	Autophagy,PI3K	3-Methyladenine (3-MA)
	GSK-3	SB216763
	GSK-3	AR-A014418
	IκB/IKK,PDK	BX-795
	mTOR	Temsirolimus (CCI-779, NSC 683864)
	mTOR	Everolimus (RAD001)
	mTOR	KU-0063794
	mTOR	WYE-354
	mTOR	AZD8055
	mTOR	Sapanisertib (INK 128, MLN0128)
	mTOR,PI3K	Omipalisib (GSK2126458, GSK458)
	PI3K	Pictilisib (GDC-0941)
	PI3K	ZSTK474
	PI3K	YM201636
	PI3K	PIK-93
	PI3K	Idelalisib (CAL-101, GS-1101)
	PI3K	Buparlisib (BKM120, NVP-BKM120)
	PI3K	CH5132799
	S6 Kinase	PF-4708671
**Proteases**	Beta Amyloid,Gamma-secretase	DAPT (GSI-IX)
	Beta Amyloid,Gamma-secretase	MK-0752
	Gamma-secretase	Dibenzazepine (YO-01027)
	Proteasome	Ixazomib Citrate (MLN9708)
**Protein Tyrosine Kinase**	ALK,c-Met	Crizotinib (PF-02341066)
	c-Kit,CSF-1R,VEGFR	OSI-930
	c-Kit,FLT3,PDGFR	Amuvatinib (MP-470)
	c-Kit,PDGFR,VEGFR	Axitinib
	c-Kit,PDGFR,VEGFR	Tivozanib (AV-951)
	c-Kit,PDGFR,VEGFR	Telatinib
	c-Met	PHA-665752
	c-Met	SU11274

	c-Met	PF-04217903
	c-Met	JNJ-38877605
	c-Met,VEGFR	BMS-794833
	CSF-1R,PDGFR,VEGFR	Linifanib (ABT-869)
	EGFR	Gefitinib (ZD1839)
	EGFR	WZ4002
	EGFR	Pelitinib (EKB-569)
	EGFR	Dacomitinib (PF299804, PF299)
	EGFR,HER2,VEGFR	AEE788 (NVP-AEE788)
	EGFR,mTOR	Chrysophanic Acid
	FGFR,PDGFR,VEGFR	Nintedanib (BIBF 1120)
	IGF-1R	Linsitinib (OSI-906)
	IGF-1R	GSK1904529A
	PDGFR	Crenolanib (CP-868596)
	TAM Receptor,c-Met	BMS-777607
	Tie-2	Tie2 kinase inhibitor
	VEGFR	Vatalanib (PTK787) 2HCl
	VEGFR	Lenvatinib (E7080)
	VEGFR,PDGFR,c-Kit	Motesanib Diphosphate (AMG-706)
**Stem Cells & Wnt**	Hedgehog/Smoothened	Vismodegib (GDC-0449)
	Hedgehog/Smoothened	Sonidegib (Erismodegib, NVP-LDE225)
	Hedgehog/Smoothened	SANT-1
	Wnt/beta-catenin	XAV-939
**TGF-beta/Smad**	PKC	Enzastaurin (LY317615)
	PKC	Sotrastaurin
	TGF-beta/Smad	SB431542
	TGF-beta/Smad	SB525334
	TGF-beta/Smad	Galunisertib (LY2157299)

Detailed information of 247 anti-cancer compounds included in the Cambridge anti-cancer compound library.

Supplementary Information 8

	AsPC-1 (μM)			BxPC-3 (μM)		
	0.1	1	10	0.1	1	10
**Crenolanib**	0.9649628	0.9055965	0.9268111	0.9514296	0.9170391	0.9654802
**Crizotinib**	0.9707937	0.9324513	0.9473687	0.9564772	0.9369009	0.9736181
**Fedratinib**	0.9668614	0.903161	0.9282085	0.9506153	0.9649385	0.9785882
**GSK690693**	0.9969551	0.935966	0.9398508	0.9599704	0.9143763	0.9642274
**PD173074**	0.9996571	0.9736348	0.9308127	0.9535848	0.9289512	0.9699826
**SGI-1776 free base**	0.9681087	0.9484474	0.9501454	0.9624862	0.9358225	0.9814099
**Trametinib**	0.9870314	0.9464467	0.9123254	0.9698686	0.9262605	0.9711223

Combination index (CI) of compounds obtained from drug screening analysis which demonstrated synergistic effect (CI<1) with NCK at all three log doses (0.1, 1, 10 μM) of NCK.

Supplementary Information 9

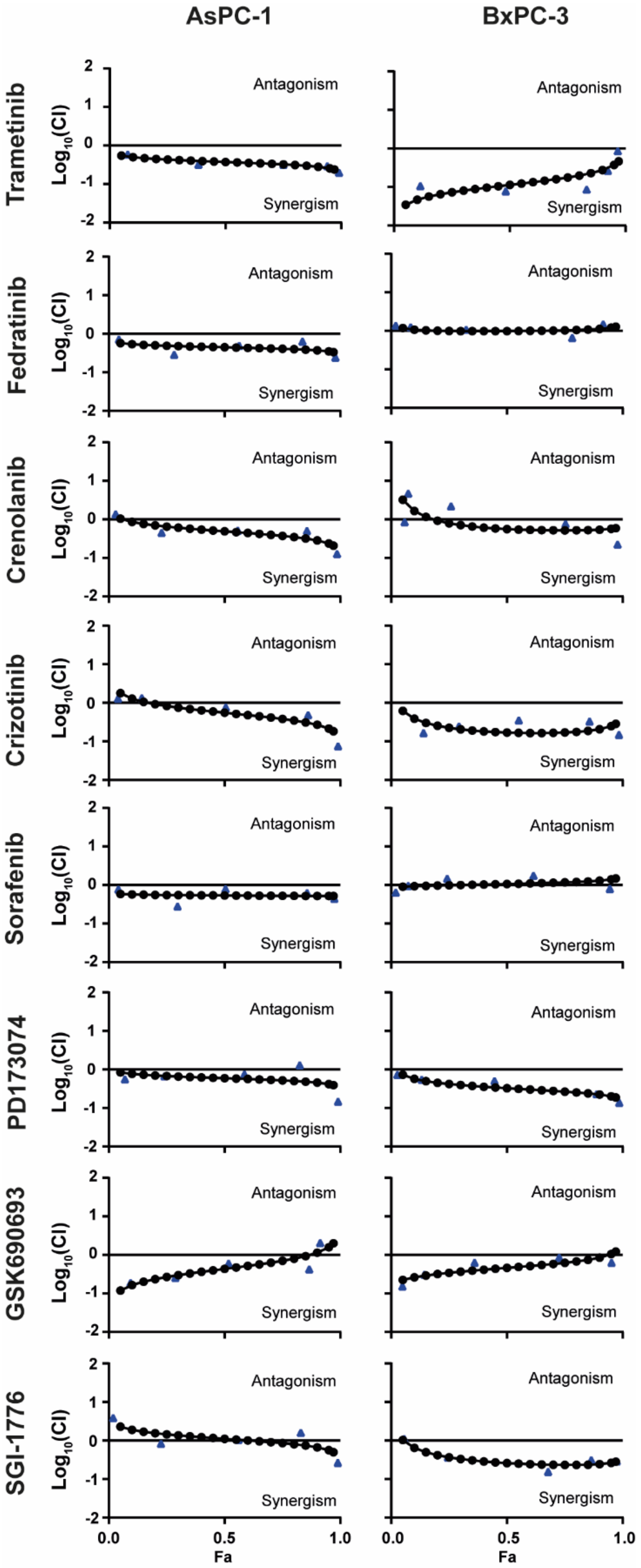

The logarithmic combination index (CI) value of NCK and Trametinib in AsPC-1 and BxPC-3 cells was determined using the Chou-Talalay method (http://www.combosyn.com). CI value indicates: <1 synergism; =1 additive; >1 antagonism.

Supplementary Information 10

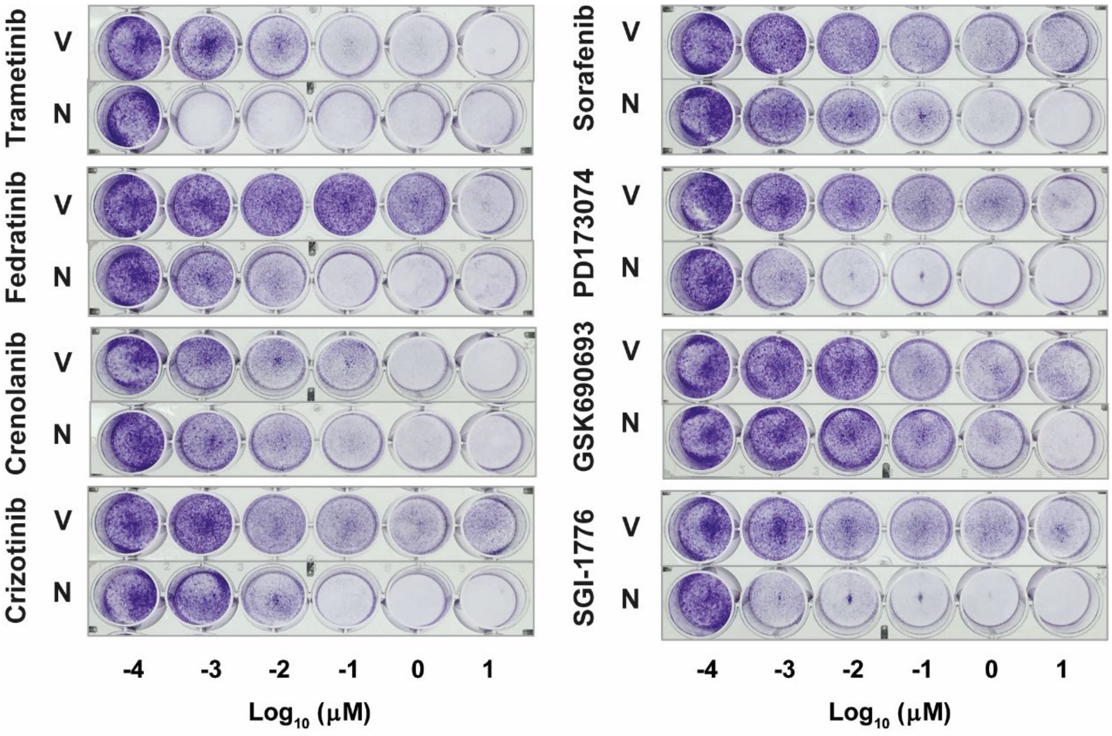

Crystal violet staining of foci in colonies of AsPC-1 cell after exposure to 8 compounds synergistic with NCK in the screening of AsPC-1 cell.

Supplementary Information 11

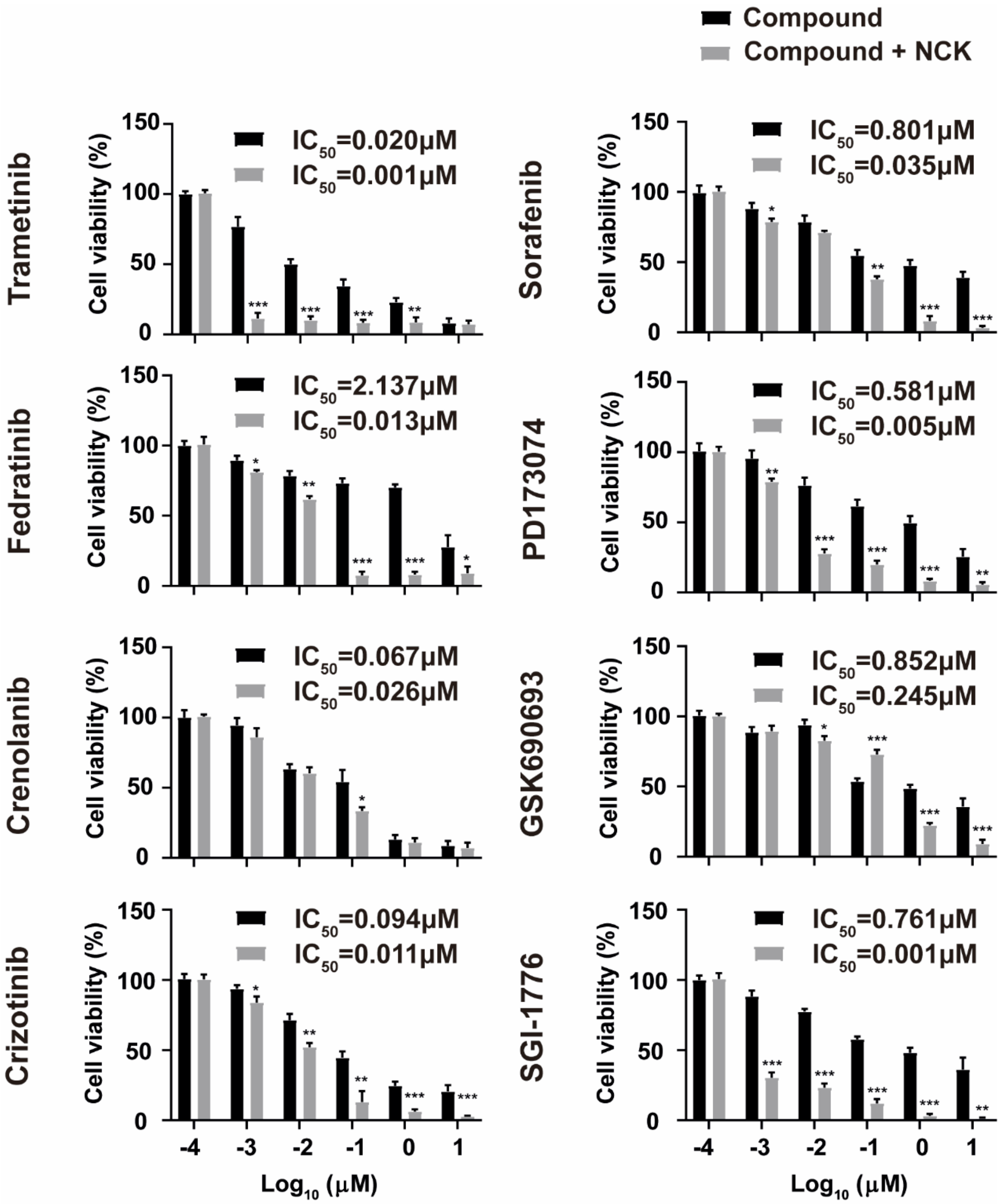

Quantification of crystal violet staining of foci in colonies of AsPC-1 cell was performed by eluting with methanol.

Supplementary Information 12

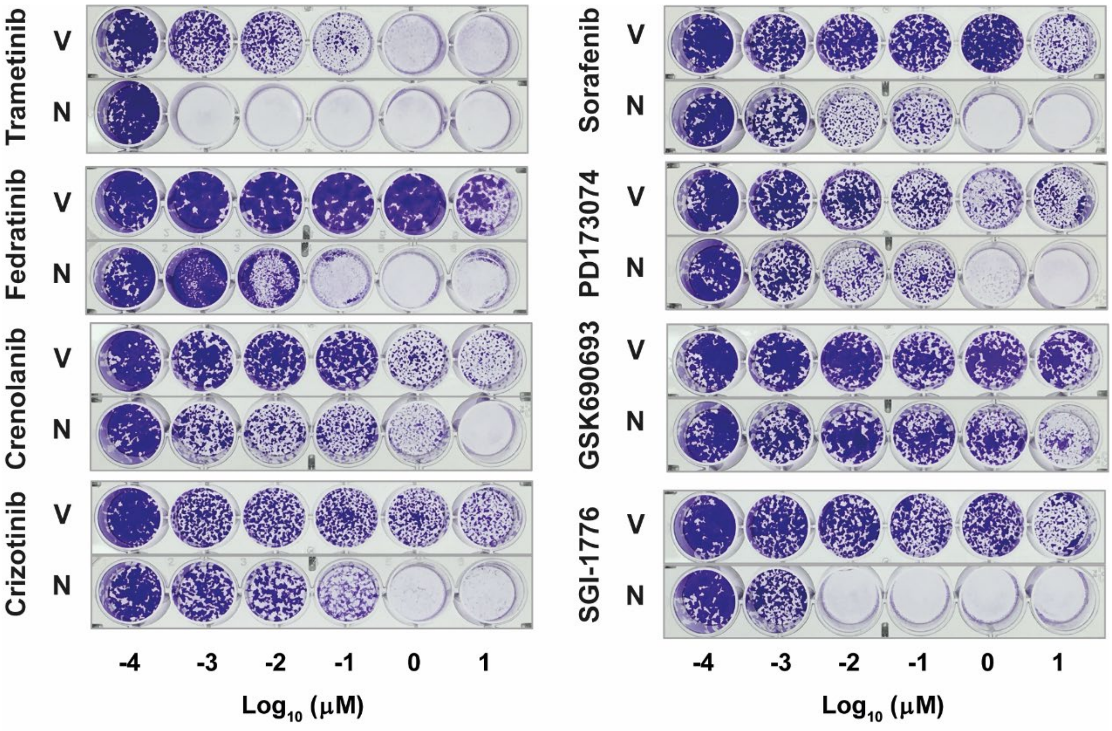

Crystal violet staining of foci in colonies of BxPC-3 cell after exposure to 8 compounds synergistic with NCK in the screening of BxPC-3 cell.

Supplementary Information 13

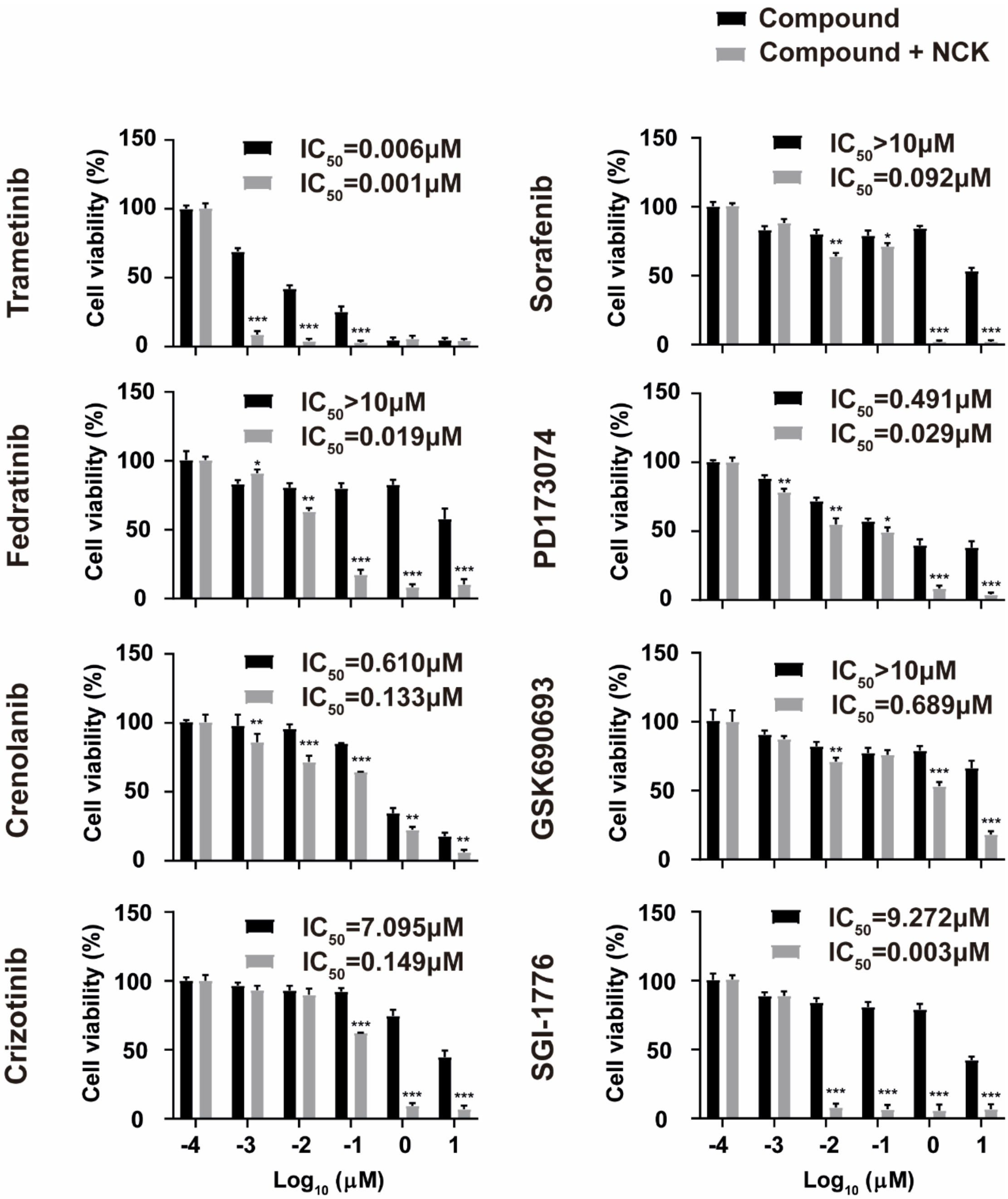

Quantification of crystal violet staining of foci in colonies of BxPC-3 cell was performed by eluting with methanol.

Supplementary Information 14

	Trametinib (T)	Selumetinib (S)	Binimetinib (B)
**Chemical structure**	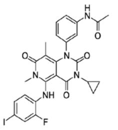	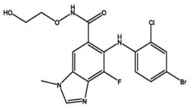	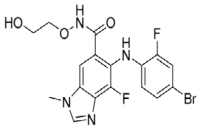
**Synonym**	GSK1120212, JTP-74057	AZD6244, ARRY-142886	MEK162, ARRY-438162
**Developer/Owner**	NOVARTIS	ASTRAZENECA	ARRAY BIOPHARMA INC
**Molecular weight (MW)**	615.39	457.68	441.23
**Target**	MEK1/2	MEK1/2	MEK1/2
***In vitro* IC50 for MEK (nM)**	0.7 (MEK1) 0.9 (MEK2)	14	12
**Cancer**	Melanoma<br>Non-Small Cell Lung Cancer<br>Thyroid Cancer	Neurofibroma	Melanoma
**Approval/development status**	Approved by US FDA (05/2013)	Approved by US FDA (4/2020)	Approved by US FDA (06/2018)
**Single-Agent dosage**	2 mg PO QD	25 mg/m^2 PO BID	45 mg PO BID

Detailed information of MEK inhibitors Trametinib (T), Selumetinib (S) and Binimetinib (B) used in current study.

Supplementary Information 15

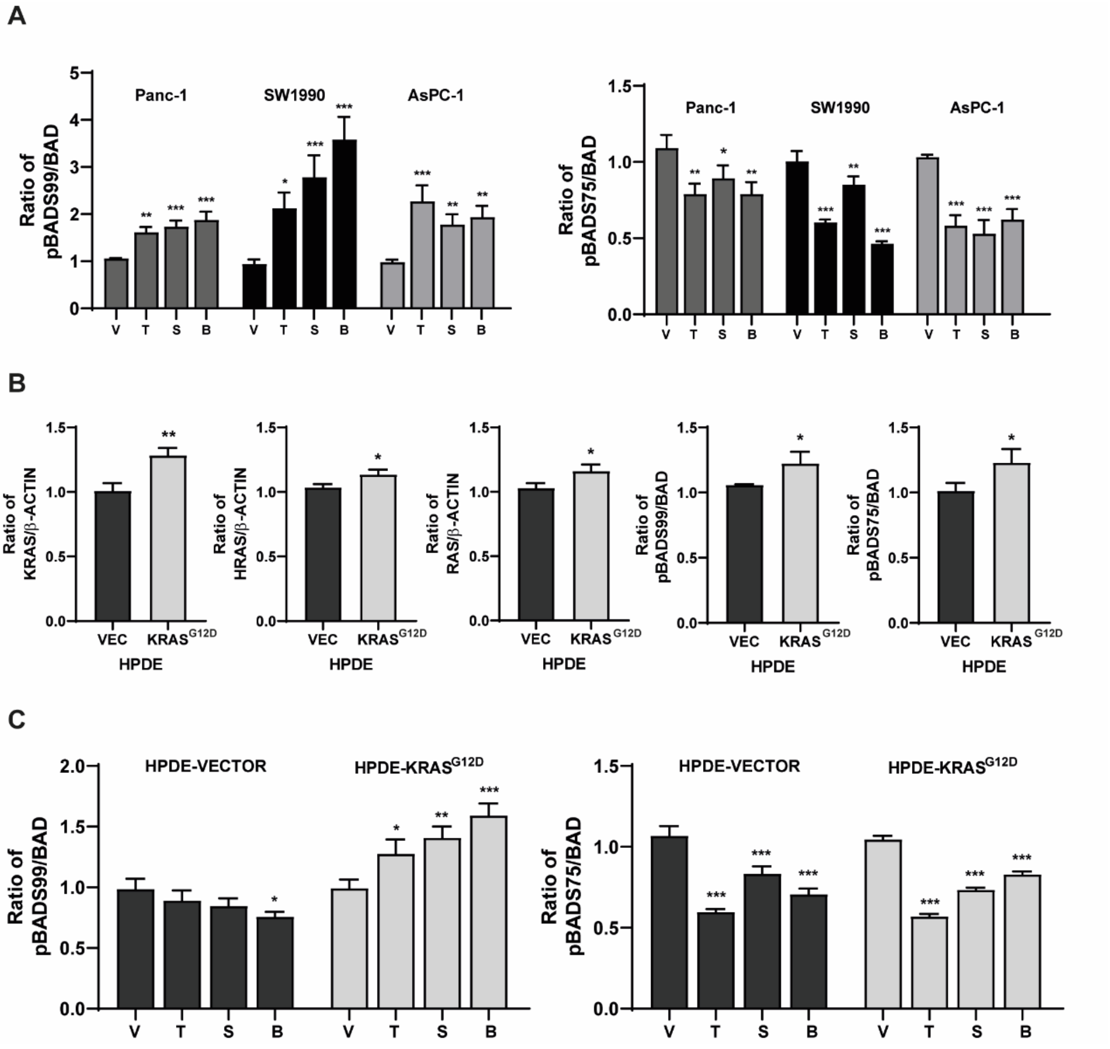

A. Densitometric analysis of western blots in Figure 5B. PDAC cells were treated with MEK inhibitors Trametinib (T), Selumetinib (S) or Binimetinib (B). Densitometric analysis of protein blots was determined using ImageJ software (https://imagej.nih.gov/ij/). Statistical changes were assessed by using ANOVA. $*P < 0.05$, $**P < 0.01$, and $***P < 0.001$.

B. Densitometric analysis of western blots in Figure 5D. Basal protein expressions of HPDE vector (HPDE-VEC) and HPDE $KRAS^{G12D}$ (HPDE-$KRAS^{G12D}$) cells were evaluated. Densitometric analysis of protein blots was determined using ImageJ software (https://imagej.nih.gov/ij/). Statistical changes were assessed by using ANOVA. $*P < 0.05$, $**P < 0.01$, and $***P < 0.001$.

C. Densitometric analysis of western blots in Figure 5E. HPDE cells were treated with MEK inhibitors Trametinib (T), Selumetinib (S) or Binimetinib (B). Densitometric analysis of protein blots was determined using ImageJ software (https://imagej.nih.gov/ij/). Statistical changes were assessed by using ANOVA. $*P < 0.05$, $**P < 0.01$, and $***P < 0.001$.

Supplementary Information 16

A

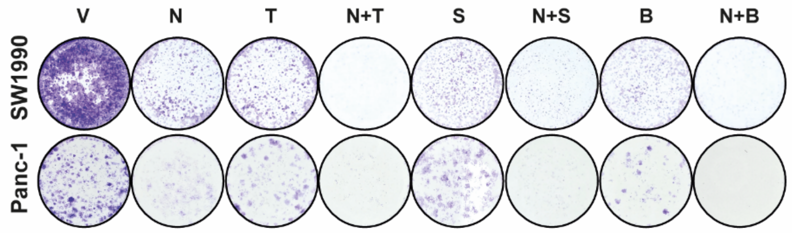

B

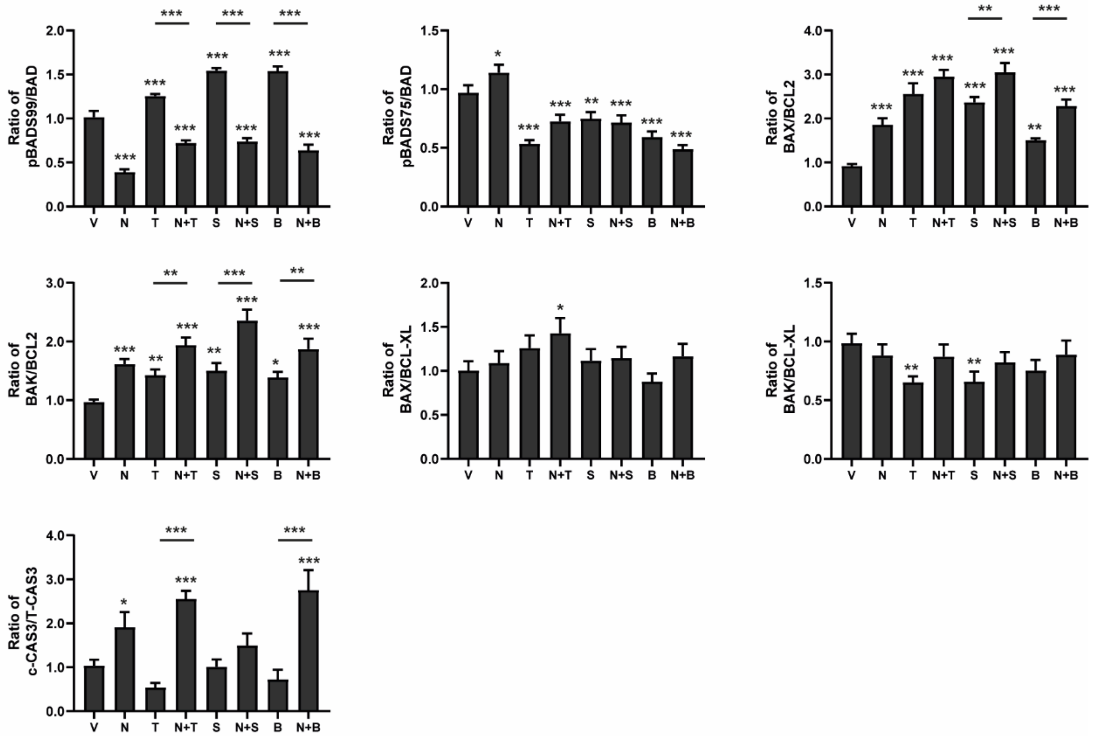

C

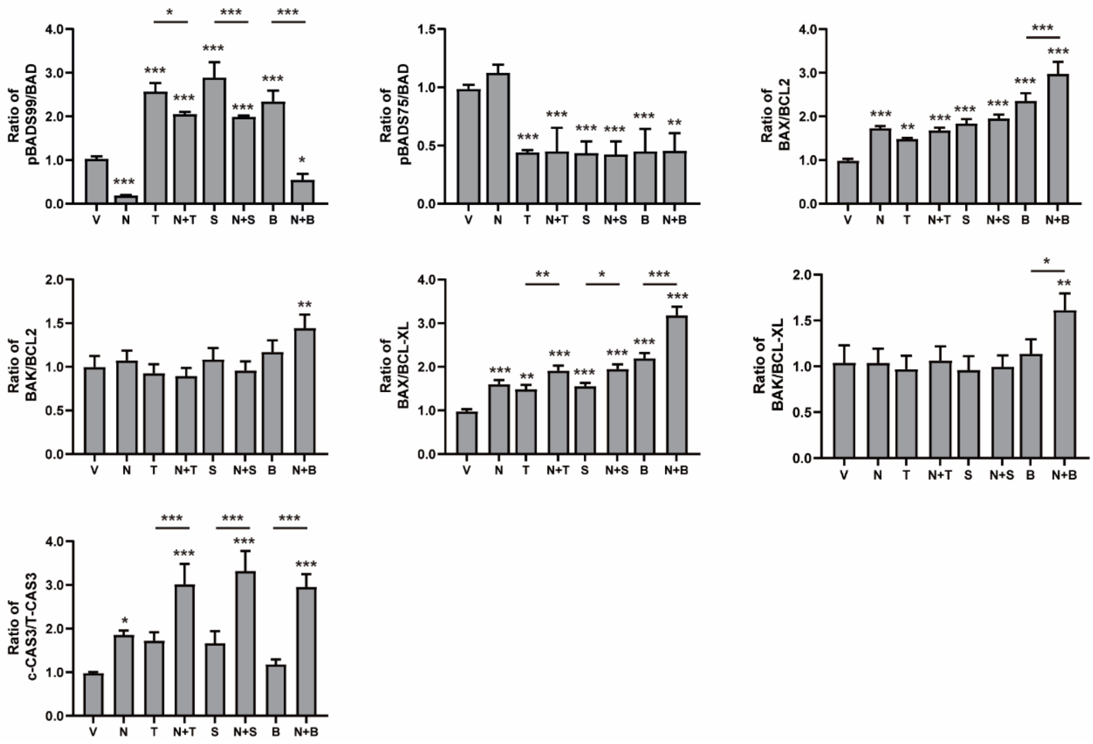

A. Crystal violet staining of foci in colonies generated by PDAC cells after exposure to NCK (N), Trametinib (T), Selumetinib (S), Binimetinib (B) or combinations. Cell viability was measured by eluting the crystal violet with methanol and detect absorbance at 595nm using microplate reader (Tecan Spark®, Switzerland). Data represent means ± SD (n=3). $^*P < 0.05$, $^{**}P < 0.01$, and $^{***}P < 0.001$.

B. Densitometric analysis of western blots in Figure 6D. SW1990 cells were treated with NCK (N), Trametinib (T), Selumetinib (S), Binimetinib (B) or combinations. Densitometric analysis of protein blots was determined using ImageJ software (https://imagej.nih.gov/ij/). Statistical changes were assessed by using ANOVA. *P < 0.05, **P < 0.01, and ***P < 0.001.

C. Densitometric analysis of western blots in Figure 6D. Panc-1 cells were treated with NCK (N), Trametinib (T), Selumetinib (S), Binimetinib (B) or combinations. Densitometric analysis of protein blots was determined using ImageJ software (https://imagej.nih.gov/ij/). Statistical changes were assessed by using ANOVA. *P < 0.05, **P < 0.01, and ***P < 0.001.

Supplementary Information 17

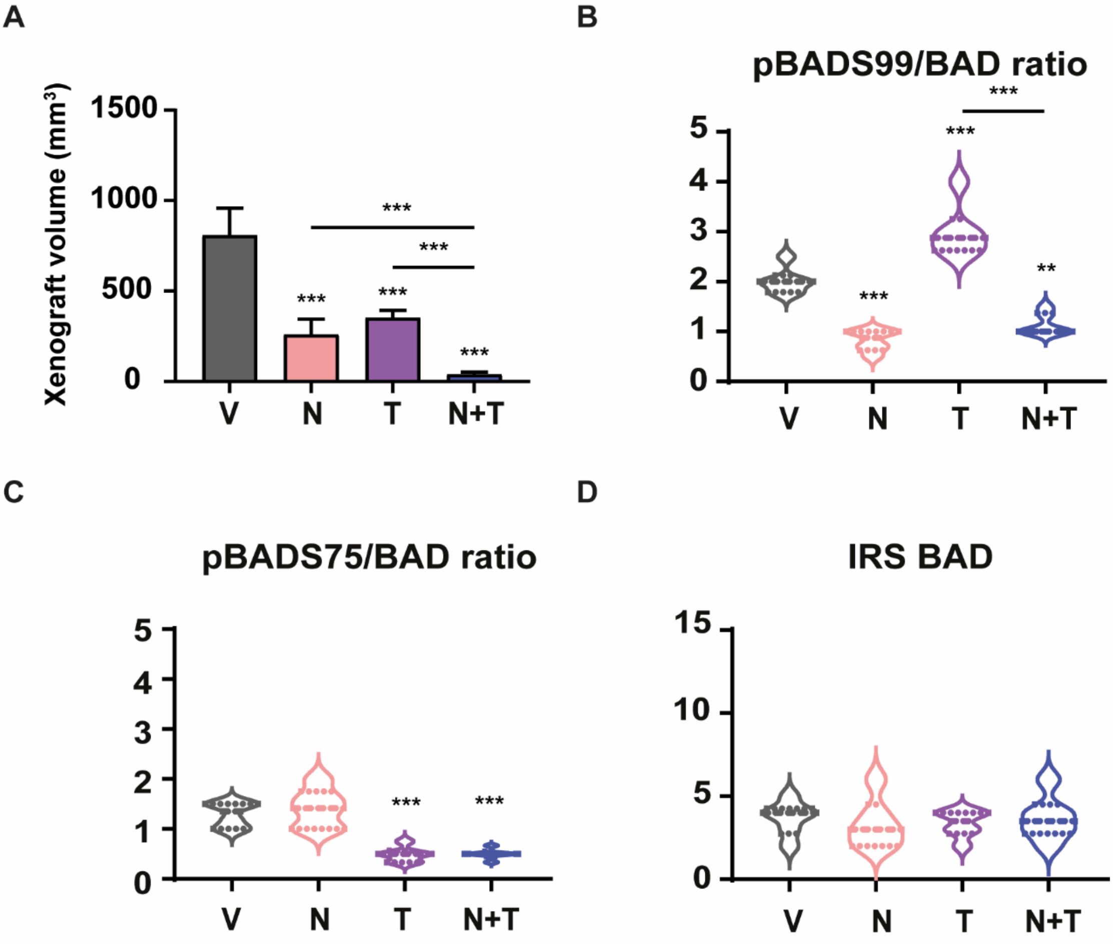

A. Mean xenograft volume of each treatment group after sacrifice at the end of 18th day. Data represent means ± SD (n=6). *$P < 0.05$, **$P < 0.01$, and ***$P < 0.001$.

B. Ratio of pBADS99/BAD IRS in xenografts. IRS scoring method is described in materials & methods. Data represent means ± SD. *$P < 0.05$, **$P < 0.01$, and ***$P < 0.001$.

C. Ratio of pBADS75/BAD IRS in xenografts. IRS scoring method is described in materials & methods. Data represent means ± SD. *$P < 0.05$, **$P < 0.01$, and ***$P < 0.001$.

D. IRS scoring of BAD in xenografts. IRS scoring method is described in materials & methods. Data represent means ± SD. *$P < 0.05$, **$P < 0.01$, and ***$P < 0.001$.

Supplementary Information 18

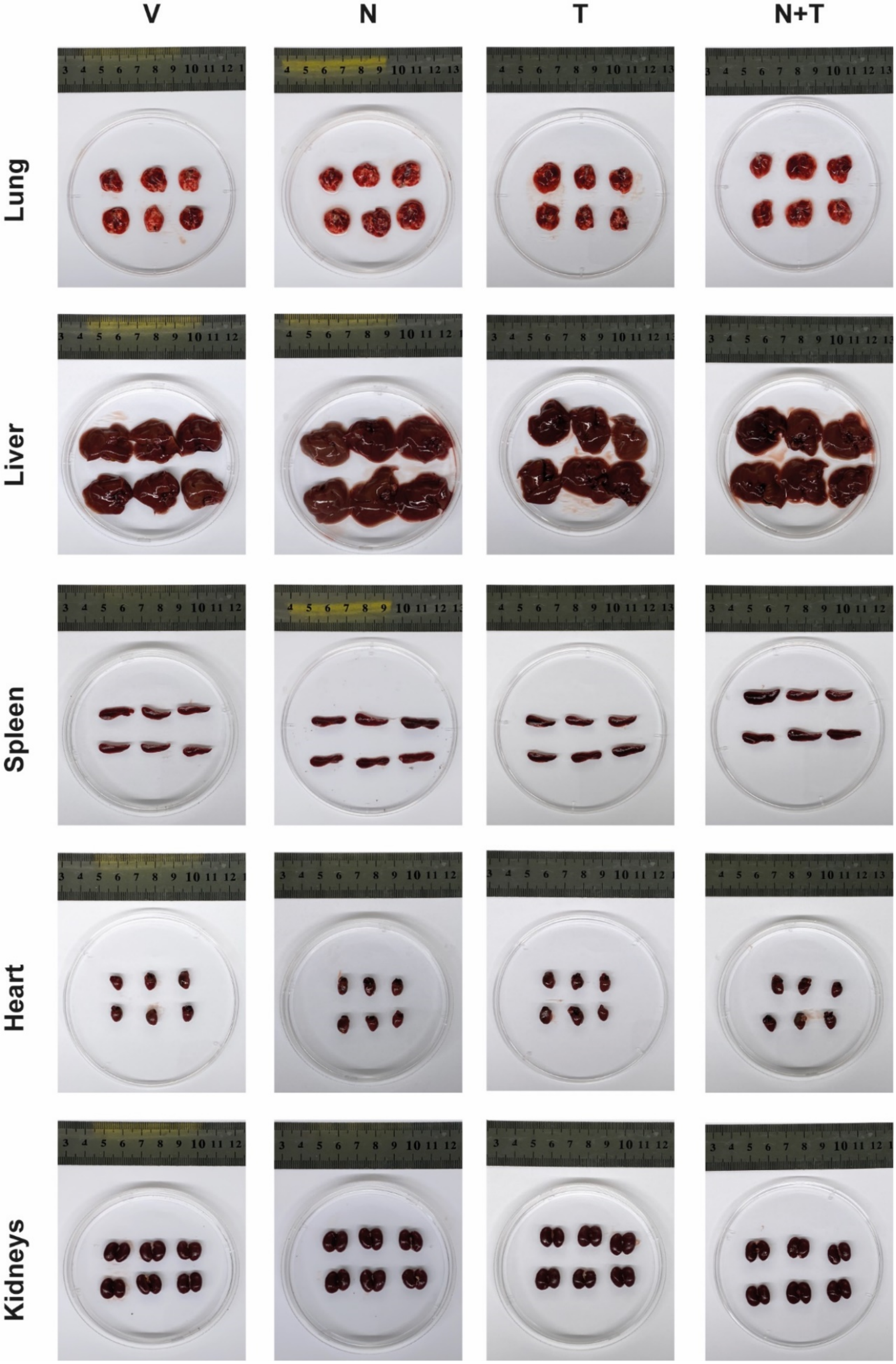

Photographs of vital organs of mice treated with vehicle (V), NCK (N), Trametinib (T) or NCK+Trametinib (N+T) after sacrifice at the end of 18th day.

Supplementary Information 19

	V	N	T	N+T	*P*-value
**Relative lung weight (%)**	1.28±0.13	1.36±0.23	1.21±0.22	1.49±0.15	0.130
**Relative liver weight (%)**	7.95±0.48	8.38±0.33	7.56±0.75	7.45±0.74	0.095
**Relative spleen weight (%)**	0.50±0.09	0.60±0.13	0.49±0.06	0.60±0.17	0.300
**Relative heart weight (%)**	0.69±0.05	0.82±0.11	0.83±0.14	0.92±0.15	0.063
**Relative kidney weight (%)**	2.20±0.13	2.15±0.14	2.15±0.19	2.08±0.18	0.690

Changes in relative lung, liver, spleen, heart and kidney weights (%, organ body index) of BALB/c-nude mice administrated with vehicle (V), NCK (N), Trametinib (T) or NCK+Trametinib (N+T) during the combination study at the end of 18th day. Data represent means ± SD.

Supplementary Information 20

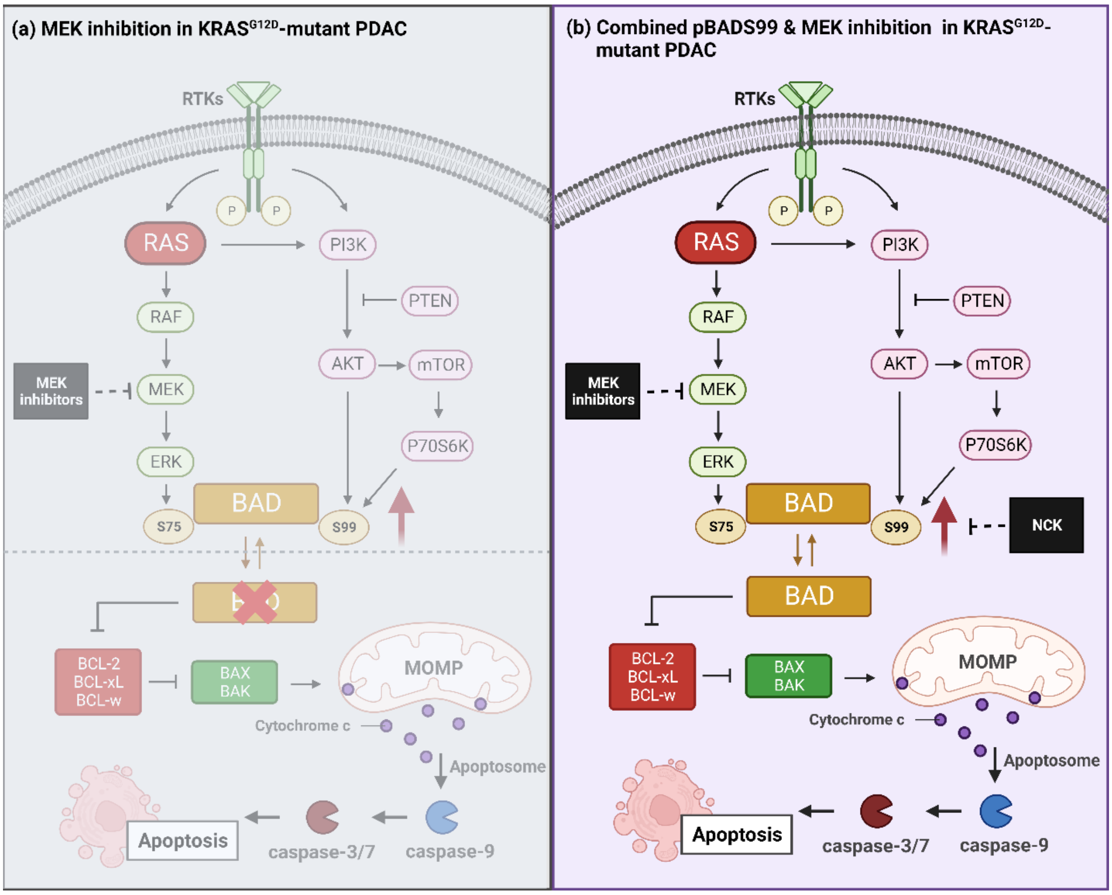

Graphical summary of the study.

Supplementary Information 21

	**CDS sequence**
**hBAD**	ATGTTCCAGATCCCAGAGTTTGAGCCGAGTGAGCAGGAA<br>GACTCCAGCTCTGCAGAGAGGGGCCTGGGCCCCAGCCCC<br>GCAGGGGACGGGCCCTCAGGCTCCGGCAAGCATCATCGC<br>CAGGCCCCAGGCCTCCTGTGGGACGCCAGTCACCAGCAG<br>GAGCAGCCAACCAGCAGCAGCCATCATGGAGGCGCTGGG<br>GCTGTGGAGATCCGGAGTCGCCACAGCTCCTACCCCGCG<br>GGGACGGAGGACGAAGGGATGGGGGAGGAGCCCAG<br>CCCCTTTCGGGGCCGCTCGCGCTCGGCGCCCCCAACCTC<br>TGGGCAGCACAGCGCTATGGCCGCGAGCTCCGGAGGATG<br>AGTGACGAGTTTGTGGACTCCTTTAAGAAGGGACTTCCTC<br>GCCCGAAGAGCGCGGGCACAGCAACGCAGATGCGGCAA<br>AGCTCCAGCTGGACGCGAGTCTTCCAGTCCTGGTGGGATC<br>GGAACTTGGGCAGGGGAAGCTCCGCCCCCTCCCAGTGA
**hBAD S99A**	ATGTTCCAGATCCCAGAGTTTGAGCCGAGTGAGCAGGAA<br>GACTCCAGCTCTGCAGAGAGGGGCCTGGGCCCCAGCCCC<br>GCAGGGGACGGGCCCTCAGGCTCCGGCAAGCATCATCGC<br>CAGGCCCCAGGCCTCCTGTGGGACGCCAGTCACCAGCAG<br>GAGCAGCCAACCAGCAGCAGCCATCATGGAGGCGCTGGG<br>GCTGTGGAGATCCGGAGTCGCCACAGCTCCTACCCCGCG<br>GGGACGGAGGACGAAGGGATGGGGGAGGAGCCCAG<br>CGCGTTTCGGGGCCGCTCGCGCGCGGCGCCCCCAACCT<br>CTGGGCAGCACAGCGCTATGGCCGCGAGCTCCGGAGGAT<br>GAGTGACGAGTTTGTGGACTCCTTTAAGAAGGGACTTCCT<br>CGCCCGAAGAGCGCGGGCACAGCAACGCAGATGCGGCA<br>AAGCTCCAGCTGGACGCGAGTCTTCCAGTCCTGGTGGGAT<br>CGGAACTTGGGCAGGGGAAGCTCCGCCCCCTCCCAGTGA

hBAD and hBAD-S99A CDSs of CRISPR-Cas9 homology directed repair (HDR).

Supplementary Information 22

Antibody	Assays	Company	Catalog no.	Dilution
pBAD (Ser136)	WB	Cell Signaling Technology	4366	1:1000
pBAD (Ser136)	IHC	GeneTex	GTX50136	1:50
BAD	WB	Cell Signaling Technology	9268	1:1000
BAD	IHC	Abcam	ab32445	1:500
MKI67	IHC	Cell Signaling Technology	9449	1:200
Cleaved Caspase-3 (Asp175)	IHC	Cell Signaling Technology	9661	1:400
Cleaved Caspase-3 (Asp175)	WB	Cell Signaling Technology	9661	1:1000
Total Caspase-3	WB	Santa Cruz	7272	1:1000
RAS	WB	Cell Signaling Technology	3965	1:1000
HRAS	WB	Abcam	86696	1:1000
KRAS	WB	Santa Cruz	30	1:1000
BCL2	WB	Cell Signaling Technology	15071	1:1000
BCLXL	WB	Cell Signaling Technology	2764	1:1000
BAX	WB	Cell Signaling Technology	5023	1:1000
BAK	WB	Cell Signaling Technology	12105	1:1000
β-ACTIN	WB	Santa Cruz	SC-47778	1:2000

Antibodies used for western blot (WB) and immunohistochemistry (IHC).
