# Supplementary material for: Concurrent inhibition of pBADS99 synergistically improves MEK inhibitor efficacy in KRASG12D-mutant pancreatic ductal adenocarcinoma
Source: Cell Death Dis. 2024 Feb 26;15(2):173. doi: 10.1038/s41419-024-06551-7 (PMC10897366; doi:10.1038/s41419-024-06551-7)
Supplement: Supplementary file 2 — Supplementary File-Original Western Blot [file 41419_2024_6551_MOESM2_ESM.pdf]

Original Western Blots for

Concurrent inhibition of pBADS99 synergistically improves MEK inhibitor efficacy in KRAS^{G12D}-mutant pancreatic ductal adenocarcinoma

Yan Qin Tan[1,2,*], Bowen Sun[1,*], Xi Zhang[1,3], Shuwei Zhang[1], Hui Guo[1], Basappa Basappa[4], Tao Zhu[3,5,6], Gautam Sethi[7], Peter E. Lobie[1,3,#] and Vijay Pandey[1,#]

[1]Institute of Biopharmaceutical and Health Engineering and Tsinghua Berkeley Shenzhen Institute, Tsinghua Shenzhen International Graduate School, Tsinghua University, Shenzhen 518055, People's Republic of China.

[2]Food Science and Technology Program, Department of Life Sciences, BNU-HKBU United International College, Zhuhai 519087, Guangdong, People's Republic of China.

[3]Shenzhen Bay Laboratory, Shenzhen 518055, Guangdong, People's Republic of China.

[4]Laboratory of Chemical Biology, Department of Studies in Organic Chemistry, University of Mysore, Manasagangotri, 570006 Mysore, India.

[5]Department of Oncology, The First Affiliated Hospital of USTC, Center for Advanced Interdisciplinary Science and Biomedicine of IHM, Division of Life Sciences and Medicine, University of Science and Technology of China, Hefei, Anhui 230027, People's Republic of China.

[6]Hefei National Laboratory for Physical Sciences, University of Science and Technology of China, Hefei, Anhui 230027, People's Republic of China.

[7]Department of Pharmacology, Yong Loo Lin School of Medicine, National University of Singapore, Singapore 117600, Singapore; NUS Centre for Cancer Research, Yong Loo Lin School of Medicine, National University of Singapore, Singapore 117599, Singapore.

[*] These authors contributed equally to this work.

[#]Correspondence to:

Peter E. Lobie, email: pelobie@sz.tsinghua.edu.cn

Vijay Pandey, email: vijay.pandey@sz.tsinghua.edu.cn

A Original western blot images for Figure 1B

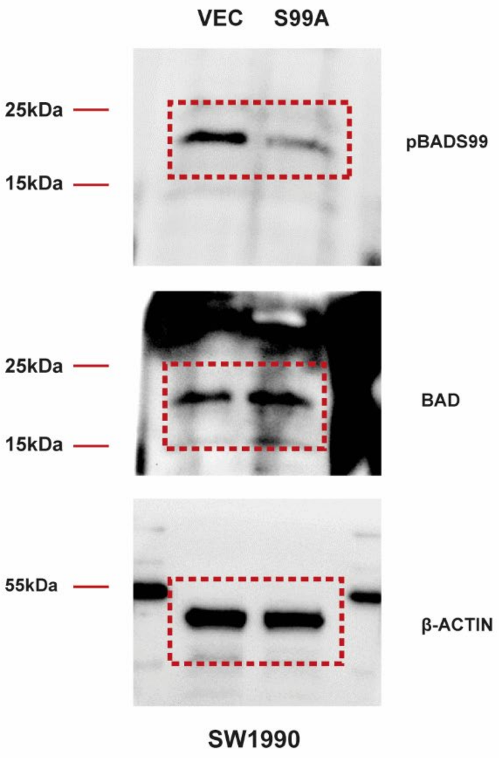

B Original western blot images for Figure 1C

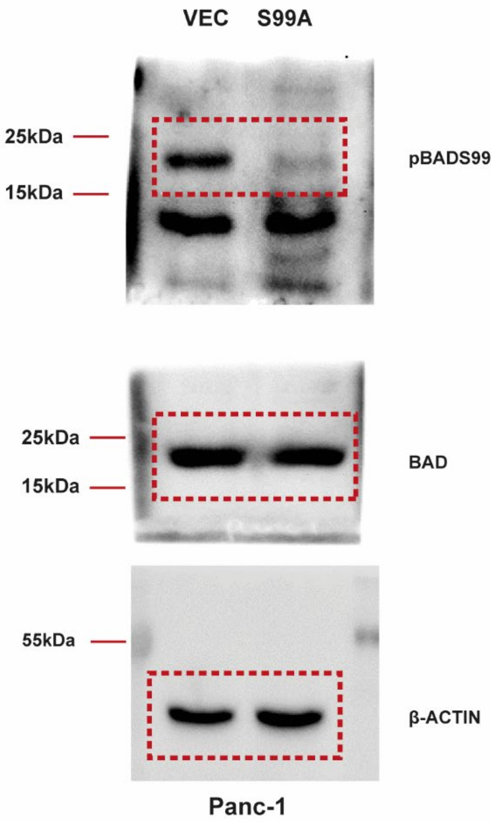

C Original western blot images for Figure 2A

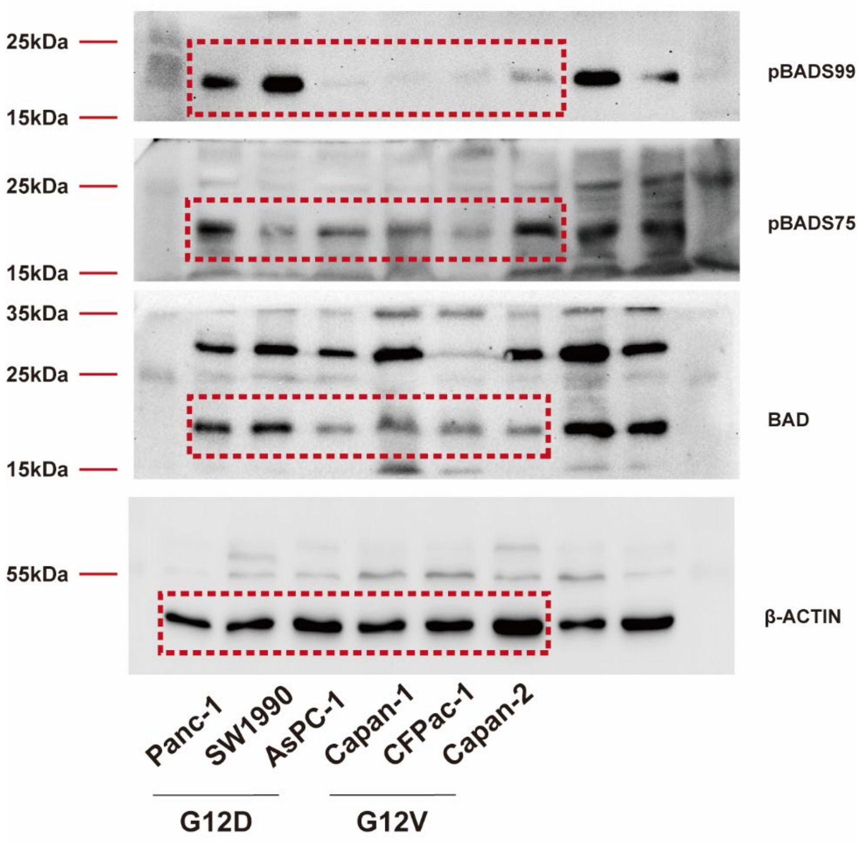

D Original western blot images for Figure 2B

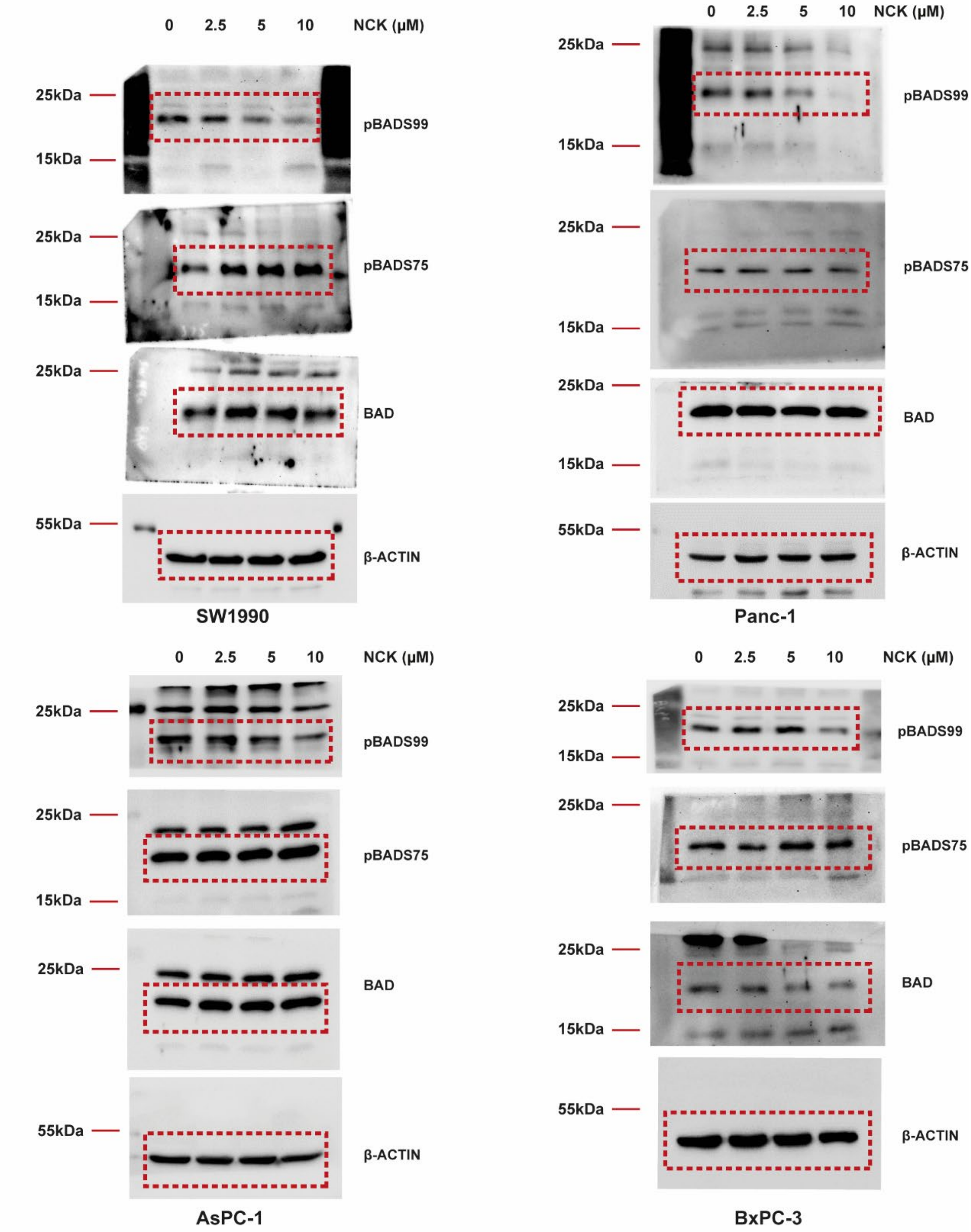

E **Original western blot images for Figure 5B**

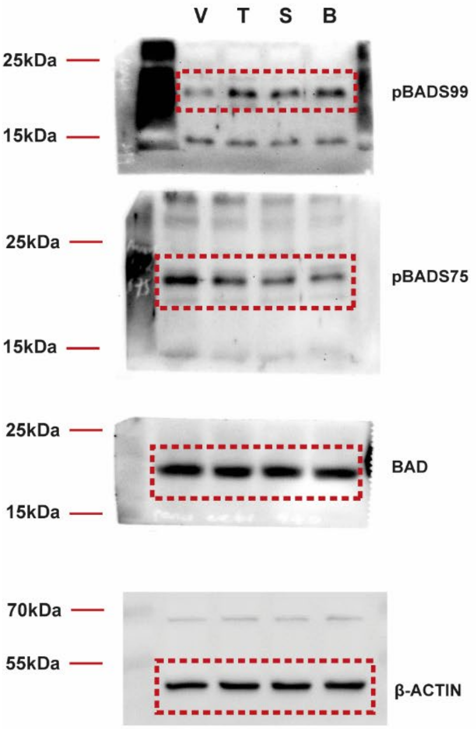

Panc-1

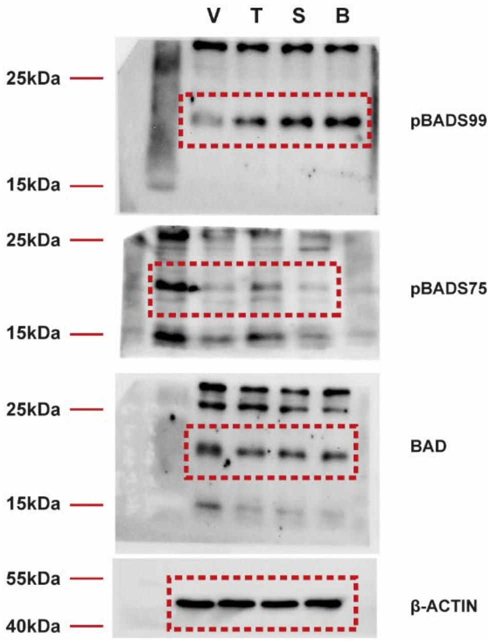

SW1990

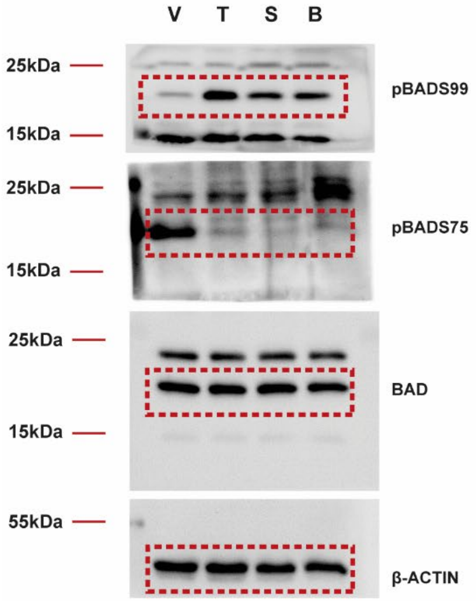

AsPC-1

F Original western blot images for Figure 5C

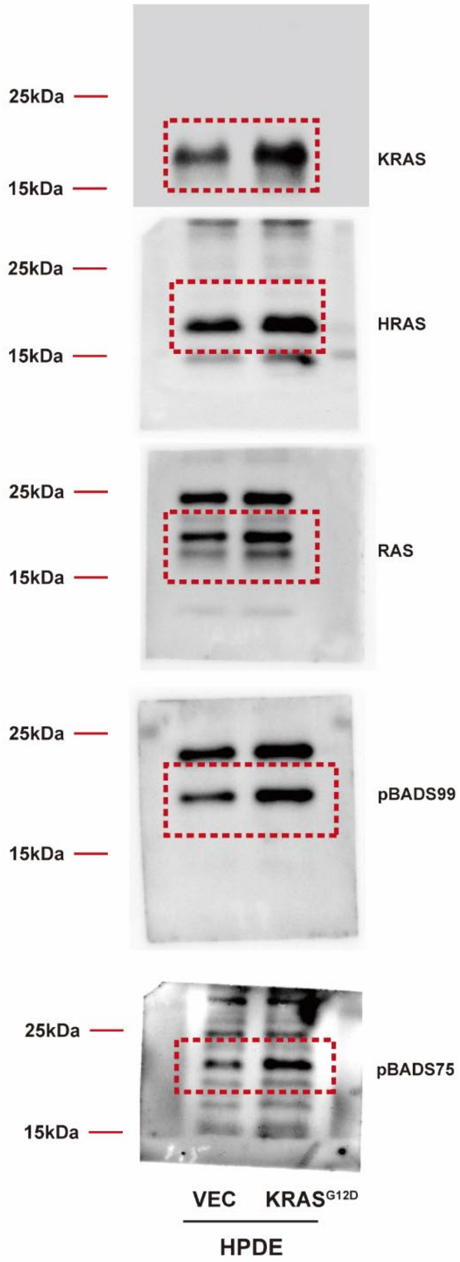

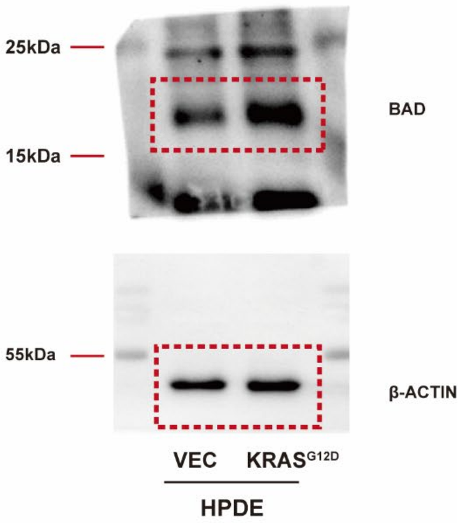

G Original western blot images for Figure 5E

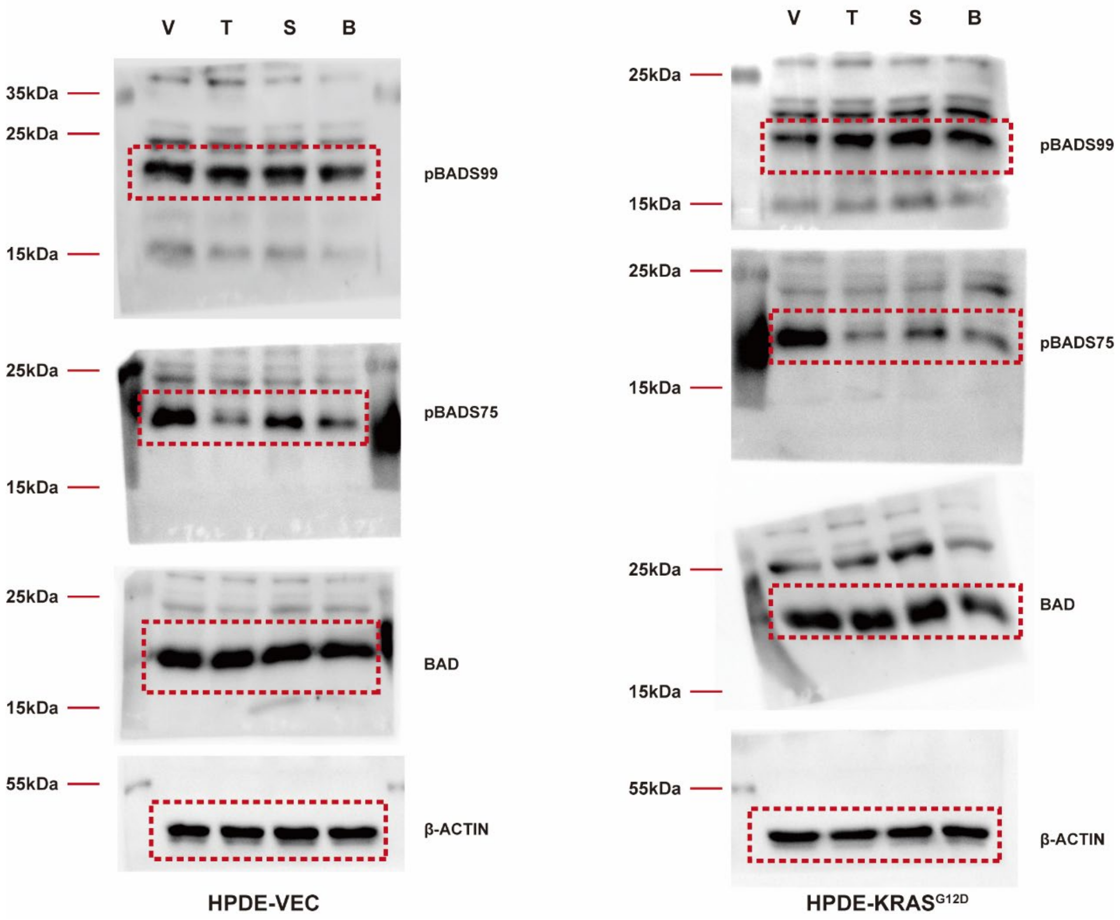

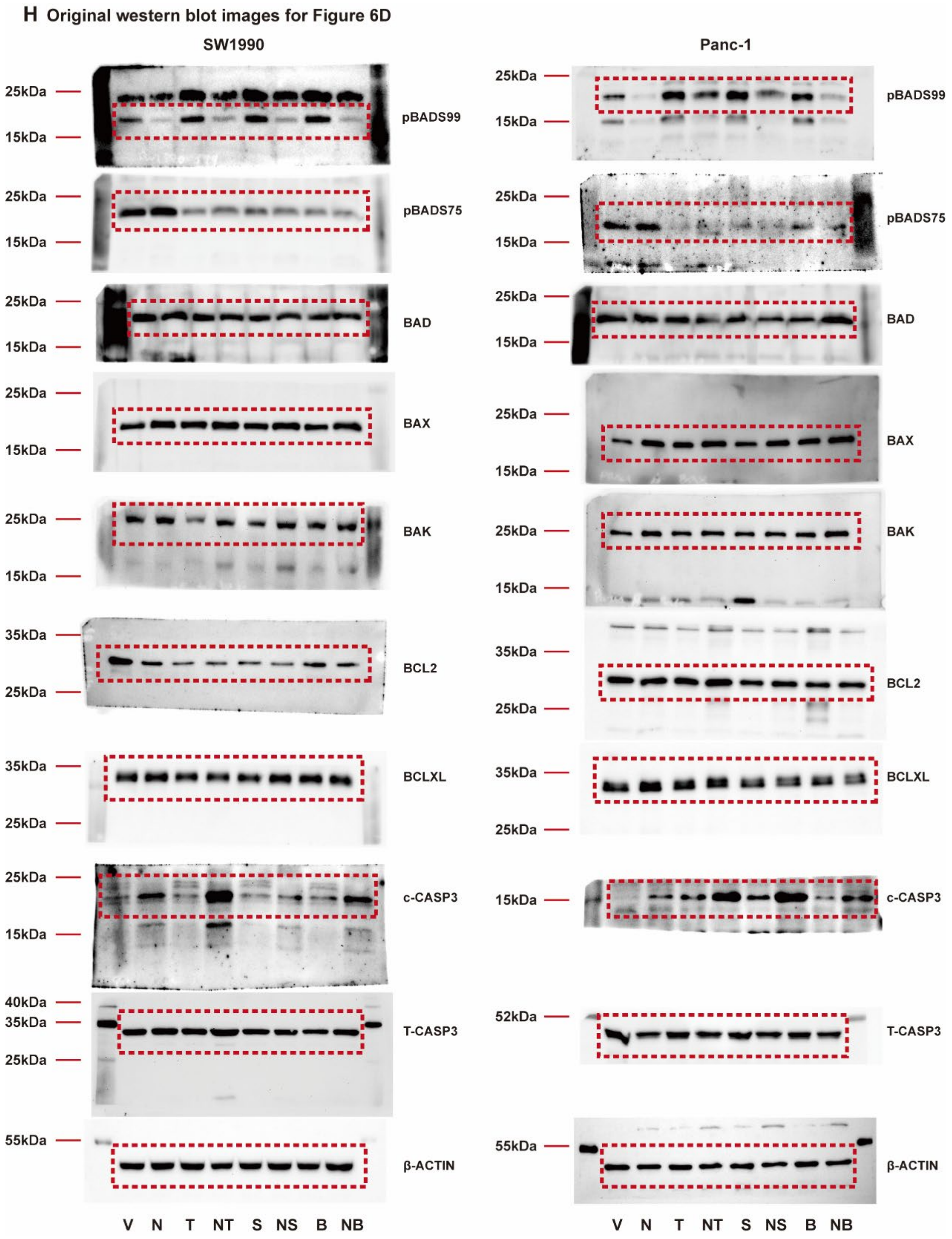

(A) Original western blot images for Figure 1B. Corresponding input controls have been provided. (B) Original western blot images for Figure 1C. Corresponding input controls have been provided. (C) Original western blot images for Figure 2A. Corresponding input controls have been provided. (D) Original western blot images for Figure 2B. Corresponding input controls have been provided. (E) Original western blot images for Figure 5B. Corresponding input controls have been provided. (F) Original western blot images for Figure 5C. Corresponding input controls have been provided. (G) Original western blot images for Figure 5E. Corresponding input controls have been

provided. (H) Original western blot images for Figure 6D. Corresponding input controls have been provided.
